# Fusion Energy and Tritium Fuel Cycle: Hydrogen Isotope Separation Technologies and Sorbent Materials

**DOI:** 10.1002/smtd.70966

**Published:** 2026-09-24

**Authors:** Pankaj Sharma, Lawrence Shere, Rosemary Brown

**Affiliations:** ^1^ United Kingdom Atomic Energy Authority Culham Campus Abingdon Oxfordshire UK

**Keywords:** absorbents, deuterium‐tritium, fusion fuel, separation, tritium exchange

## Abstract

Fusion using tritium and deuterium as the fuel has the potential for large‐scale carbon‐free energy generation but faces several key technical challenges; One of these is the need to develop a hydrogen isotope separation technique with the combined requirements of high separation and energy efficiency and low tritium inventory. Since only a small fraction of the injected fuel undergoes fusion per cycle, the mixture of hydrogen isotopes must be recycled and the isotope composition rebalanced, requiring hydrogen isotope separation. This task is technically complex because hydrogen isotopes exhibit nearly identical physicochemical properties making them difficult to separate conventionally. Cryogenic distillation currently serves as the reference technology for isotope separation but suffers from high energy demand, extreme cryogenic operating temperatures, and significant tritium hold‐up. Sorbent‐based processes have emerged as promising candidates, offering advantages in scalability, energy efficiency, and isotope selectivity. Sorbent materials that could be used in these processes have been reported to have high hydrogen isotope selectivity and so could have significant potential. This review examines recent advances in sorbent development and their integration into separation processes relevant to fusion fuel cycles, while also addressing key challenges such as stability, radiolytic degradation, and tritium exchange in organometallic porous adsorbents.

## Graphical abstract

This review surveys sorbent‐based materials for hydrogen isotope separation, a key challenge in fusion fuel processing. Different types of adsorbents are compared in terms of selectivity, kinetics, and operational practicality. Limitations of cryogenic operation and tritium compatibility are highlighted, providing guidance toward efficient, low tritium inventory isotope separation technologies for future fusion fuel processing.

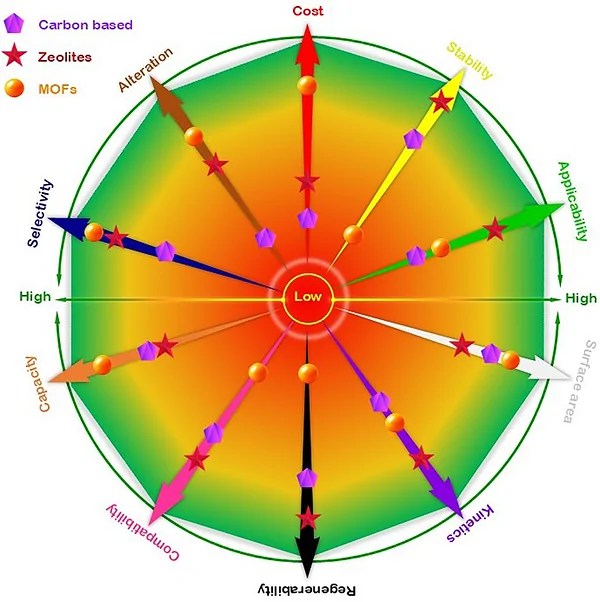

## 1 Introduction

### 1.1 The Need for Fusion Energy

With rising concerns over greenhouse gas emissions and environmental degradation, transitioning to clean energy sources has become a global priority. However, transitioning away from fossil fuels to mitigate greenhouse gas emissions poses considerable challenges in meeting the world's escalating energy needs. Renewable energy sources such as solar and wind are rapidly expanding, but tend to be intermittent, leading to instabilities in the electrical grid and in many countries, the availability of renewable energy cannot match demand [1, 2].

Fusion is a potential source of future energy that holds significant potential as a secure and abundant energy source that could contribute to long‐term energy sustainability [3, 4]. Furthermore, fusion offers distinct advantages in comparison to nuclear fission, such as minimal production of long‐lived radioactive waste, and inherent safety features. Fusion energy holds the potential to provide an inexhaustible supply of clean power by replicating the sun's fusion process, offering a continuous and reliable energy source regardless of weather conditions or geographical limitations. provided that technological challenges can be overcome. Fusion energy has witnessed substantial advancements in technology and increased funding over the past few decades, with backing from both governments and private investors (Figure 1a), emerging as a leading contender for future global energy solutions [5, 6].

**FIGURE 1:**
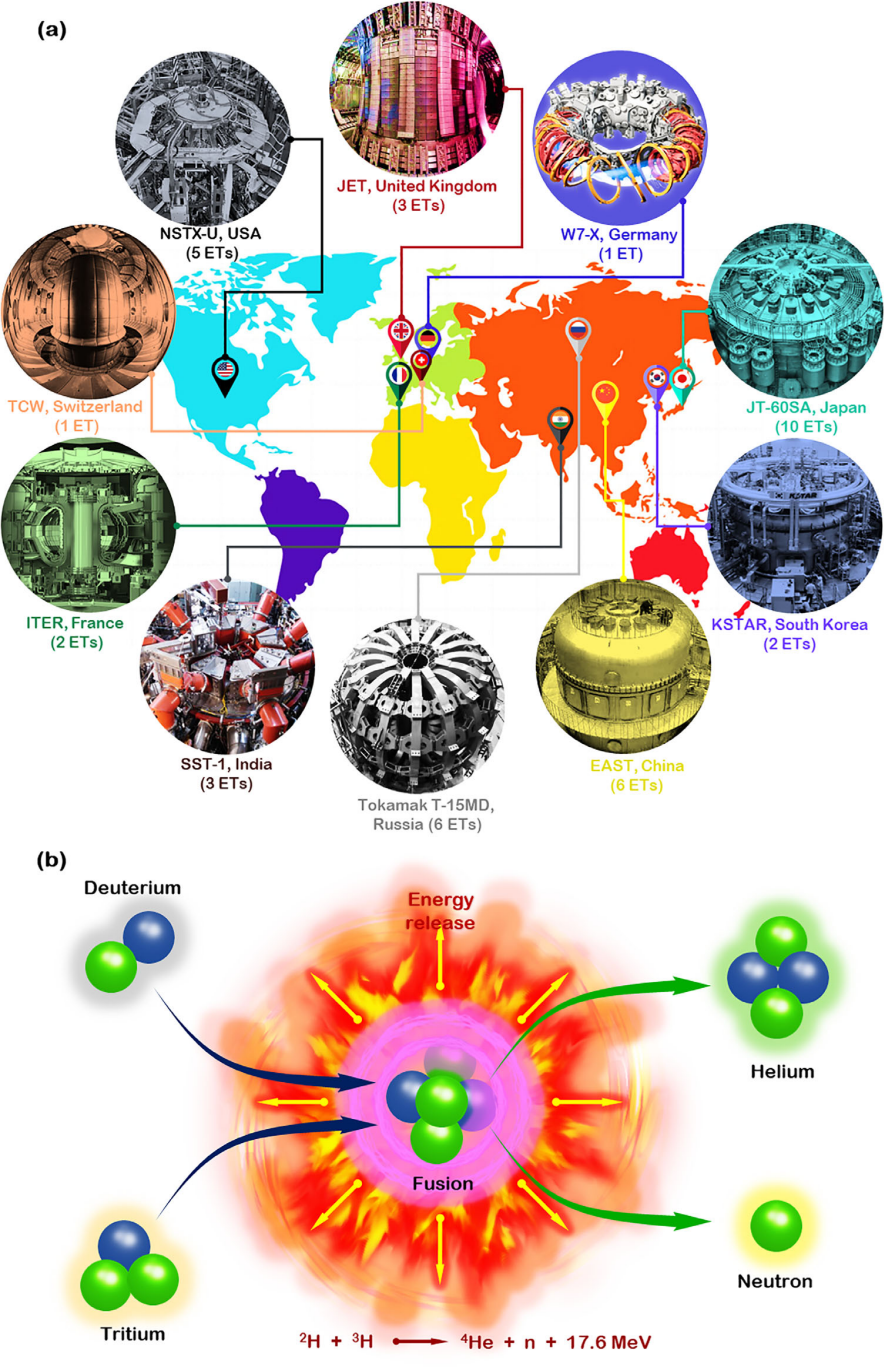
(a) Fusion for future: a global endeavour to harness fusion energy. A comprehensive look at experimental tokamaks (ETs) operating across various regions worldwide, united in their pursuit of realising fusion energy. Some of the experimental tokamaks operating and under construction: Brazil (3 ETs) [7], Canada (1 ET) [8], China (6ETs) [9], Costa Rica (1 ET) [10], Czech Republic (2 ETs) [11], Denmark (1 ET) [12], France (2ETs) [13], Germany (1ET) [14], India (3ETs) [15], Italy (1 ETs), Japan (10 ETs) [16, 17], Kazakhstan (1 ET) [18, 19], Pakistan (4 ETs) [20], Portugal (1 ET) [21], Russia (6ETs) [22], South Korea (2ETs) [23], Spain (1 ET) [24, 25], Switzerland (1ET) [26], Thailand (1 ET) [27, 28], United Kingdom (3ETs) [29], and United States of America (5 ETs) [30]. *Source*: World Survey of Fusion Devices 2022, Copyright 2022 International Atomic Energy Agency [5]. The tokamak images shown in this figure are provided solely as a brief visual overview of the respective facilities. The images were obtained from publicly available sources, and the original publication/source of some images could not be definitively identified. Image ownership and associated credits therefore remain with the respective organisations. (b) Representation of fusion reaction. Fusion, which operates on the principle of releasing energy by combining atomic nuclei rather than splitting them as in fission reactions, represents the grand engineering challenge of the twenty‐first century in harnessing clean energy.

### 1.2 Fusion Energy

Fusion is a process which powers the Sun in which lightweight atomic nuclei typically isotopes of hydrogen combine to form heavier nuclei like helium, releasing a large amount of energy. This reaction occurs at extremely high temperatures (150 million kelvin) place in a high‐energy state of matter known as plasma (Figure 1b). The challenge of achieving fusion energy is confining the fuel in this extremely hot plasma state for long enough that the fuel can react and release more energy than is provided to initially heat the plasma. The fusion of deuterium and tritium (D‐T fusion) is one of the most promising fusion reactions as given by the Lawson criterion, since the reaction rate is higher and releases more energy at lower temperatures than other fusion reactions. D‐T fusion produces nearly four times as much energy per kilogram of fuel, compared to fission (utilised in nuclear power plants) [31], and nearly four million times more energy than burning coal, oil, and gas.

It is important to note that tritium is a scarce worldwide resource [32]. Tritium is also radioactive with a half‐life of 12.3 years so there are strict limitations on the site inventory and preventing release to the environment. Fusion fuel cycles are required to recycle tritium from the reactor exhaust for re‐injection due its scarcity. All isotopologues of hydrogen need to be carefully managed to maintain purity and limit release so significant efforts are underway to develop technologies that can process reactor exhaust safely and efficiently.

## 2 Growth in Fusion Industry and Research

Though the journey toward harnessing fusion has been challenging, recent advancements in D‐T fusion signal a transformative era in fusion energy research and development. Numerous fusion projects, both public and private, are underway globally. According to the Fusion Industry Association's 2024 report on the global fusion industry, the landscape is further enriched by 45 private companies actively developing over 15 distinct fusion power concepts, signalling the emergence of a fusion industry [6, 33]. These initiatives span experimental tokamaks, stellarators, heliotrons, alternative fusion devices, demo units, and demonstration designs, at various stages of operation, construction, or planning. Most of these fusion concepts are built around DT fusion and thus require DT fuelling systems.

Researchers at the Joint European Torus (JET), operated by the Culham Centre for Fusion Energy (CCFE), have achieved a remarkable milestone in fusion energy. They released 69.26 mJ of heat within a single pulse (5.2 s), using a mere 0.21 mg of fuel over a 6‐s period [34]. This achievement is equivalent to the energy generated by burning 2 kg of coal.

This groundbreaking accomplishment at JET supersedes their own previous record of 59 megajoules with 0.17 mg of fuel achieved during a 5‐s pulse. Importantly, the scientists at JET have demonstrated their ability to consistently replicate the requisite fusion conditions across multiple experimental pulses. These recent advancements, coupled with global research, have made significant strides in fusion and plasma physics, addressing numerous scientific questions and fostering technological developments for safer and more sustainable fusion reactor operation.

Progress in JET operations are a multi‐disciplinary achievement which required vast upgrades to the tokamak and the installation of the active gas handling system (AGHS), the world's only tritium fuel cycle attached to a tokamak. This fuelling system was instrumental in achieving deuterium‐tritium (D‐T) operations and the highest energy output so far.

This progress serves as a driving force, encouraging researchers from over 50 member states of the International Atomic Energy Agency to engage in controlled fusion energy research. According to the Global Fusion Industry Report (2023), the fusion industry has now attracted over $6 billion in investment, marking a $1.4 billion increase from the previous year. Additionally, the report highlights a notable increase in the number of fusion energy‐focused private companies each year, with existing firms consistently boosting their funding levels annually (Figure 2) [6].

**FIGURE 2:**
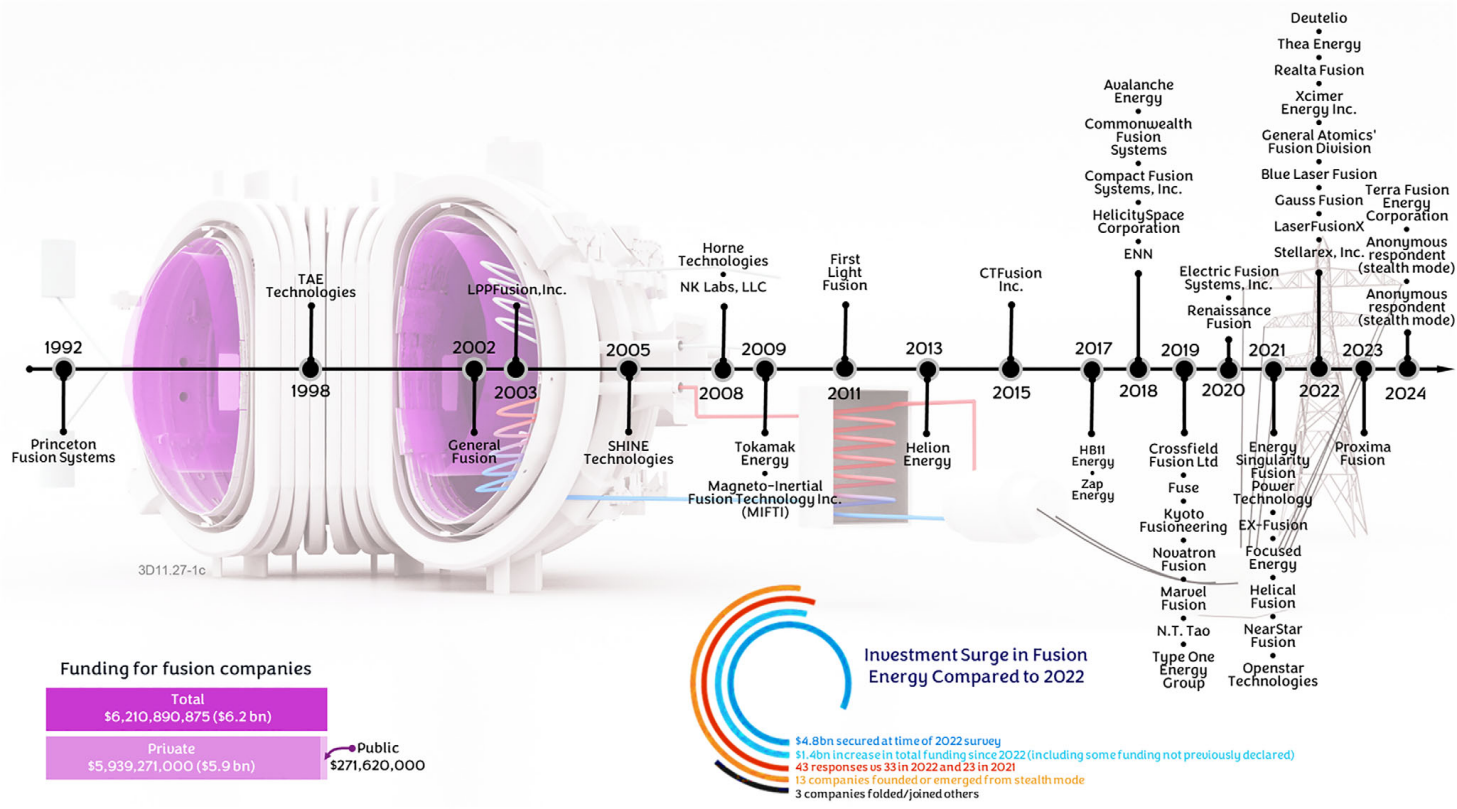
Significant private investment in fusion energy signals advancement toward sustainable energy provision. An overview of the founding years of various fusion energy enterprises, coupled with the notable surge in investment in the field, alongside funding for fusion companies, and yearly tallies of private fusion energy companies. *Source*: Fusion Industry Association: 2023 [6] and 2024 [33] Annual Global Fusion Industry Report. The background image is credited to EUROfusion, Copyright United Kingdom Atomic Energy Authority (UKAEA).

Expanding on the foundation laid by JET, the International Thermonuclear Experimental Reactor (ITER) commenced construction in France in 2013. ITER's establishment expanded international cooperation in tokamak fusion to include 35 countries, nurturing a worldwide atmosphere conducive to open scientific exploration. Furthermore, the National Ignition Facility (NIF) at Lawrence Livermore National Laboratory achieved a significant milestone last year by demonstrating a net energy gain from their fusion experiments, involving the firing of laser beams at frozen hydrogen isotopes [35].

Recent advancements have undoubtedly propelled D‐T fusion research into new territory. Nonetheless, significant challenges remain within the tritium fuelling system. Tritium is both scarce and hazardous, making it essential to carefully manage and minimise the inventories required to operate a fusion power plant. Isotope separation is highly challenging due to the nearly identical physical and chemical properties of hydrogen isotopes, which demand advanced, energy‐efficient separation technologies with high performance and throughput. The primary focus of this review article will be on the challenge of separating hydrogen isotopes and enriching deuterium and tritium in the tritium fuel cycle for their reuse. There is a focus on recent advancements in materials development and testing results with tritium as this is key for the development of sorbent‐based systems, which form the majority of separation system candidates.

## 3 Tritium Fuel Cycle

Given the minimal fraction of injected fuel reaching the plasma core and the anticipated low burn‐up rates [36, 37, 38, 39, 40], future powerplants will be required to process vastly greater quantities of tritium than the 100 g or so in JET's AGHS [41]. For instance, in the case of DEMO, which is envisioned to consume 313 g of tritium daily to yield 2 GW of energy [42, 43, 44].

The fusion reactor fuelling system is designed to recycle and reprocess the exhaust gases from the torus so that they can be reinjected. A simplified process is shown in Figure 3 [45]. It also includes the safe storage of tritium and deuterium, and the removal of tritium (detritiation) from stack exhaust streams for safe discharge. Modern designs contain tritium breeding blankets to produce more fuel in‐situ, the fuel cycle must include the extraction and management of tritium and impurities generated in breeding blanket modules.

**FIGURE 3:**
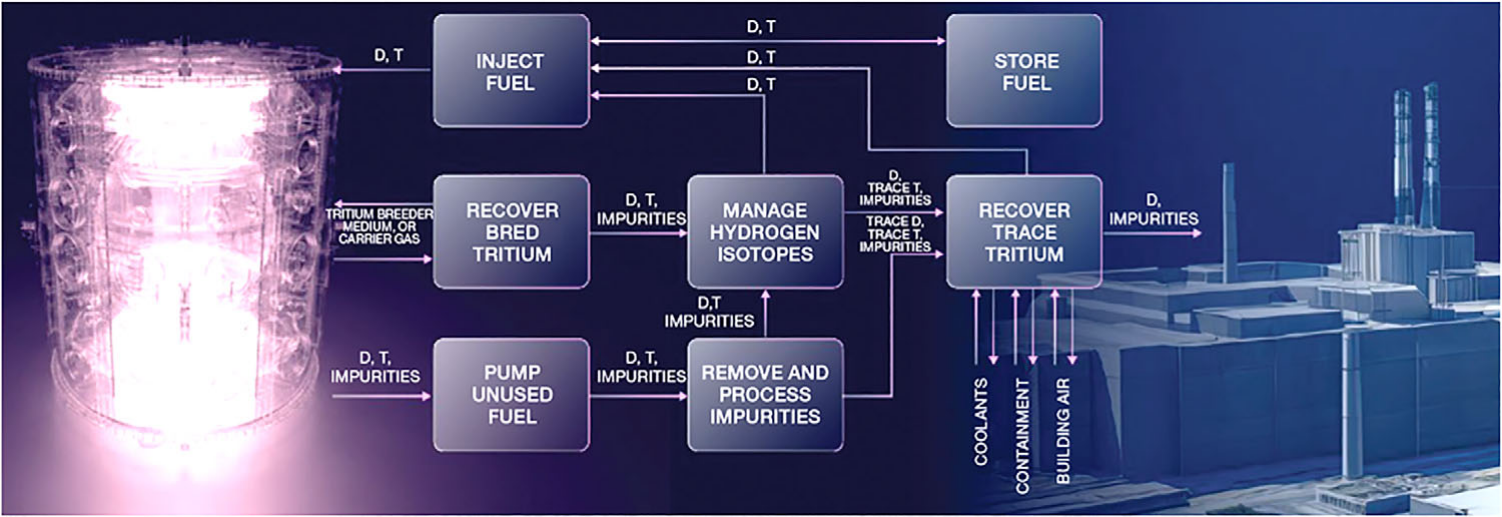
A simplified illustration of the fusion fuel cycle highlights the significance of separation and recovery procedures within the deuterium‐tritium (D‐T) fuelling cycle. As depicted in the diagram, the hydrogen isotope separation system is at the centre of the fuel cycle and is required to recycle and rebalance the fusion fuel. Image courtesy of E. Loving and T. Stroud, UKAEA. Reproduced from Ref. [45].

The tritium fuel cycle represents a significant technical challenge. Hydrogen isotope separation is required to manage isotopologue gas mixtures for both reprocessing of the D‐T fuel for reuse and exhaust detritiation. The hydrogen isotope separation system must be able to achieve high separation and energy efficiency, while maintaining a minimum tritium inventory.

Day et al. [46]. comprehensively detailed the pre‐concept design (PCD) phase of EU‐DEMO, highlighting the development of a tritium self‐sufficient three‐loop fuel cycle architecture aimed at minimising tritium inventory within the systems [42, 47, 48, 49]. This approach necessitates the continuous recirculation of gases in loops without storage, prioritising continuous technologies to prevent tritium hold‐ups at each process stage, and permits immediate utilization of tritium extracted from tritium breeding blankets. Recycling fuel directly from the reactor and bypassing the ISS system via a direct internal recycling route provides more flexibility in the operational parameters for the ISS and therefore permits use of different technologies. These technologies require optimisation as they are low readiness level and high inventory in most cases.

For all fuel cycle architectures there is a balance to be made between technologies that have been proven and technologies which can be scaled up from the current fusion test‐reactor sizes like JET. For isotope separation this is a significant issue, for example scaling up cryogenic distillation could lead to a large residence time and significant tritium hold‐up within the system which makes it difficult to implement without significant improvements and research [50]. Likewise, gas chromatography (used in AGHS) is not easily made continuous even though it produces high purity tritium [48, 51]. The fuel cycle must be a continuous process for fusion power to be viable.

## 4 Isotope Separation Techniques

The JET fusion reactor, as well as other world's major fusion reactors such as the ITER [52, 53], EU‐DEMO [54, 55], and China Fusion Engineering Experimental Reactor (CFETR) [56, 57] employs various hydrogen isotope separation processes to facilitate its operations [58]. Cryogenic distillation is currently one of the most commonly used techniques and will be used in ITER. However, it requires extremely low temperatures (24 K) and has a very high tritium inventory. Some viable alternative methods are sorbent based which include gas chromatography, thermal cycling absorption process (TCAP), pressure and temperature swing adsorption methods and membrane‐based methods [43]. A direct comparison of energy demand, tritium inventory, scalability, and technology readiness is difficult because the technologies are at different stages of development and none has yet been demonstrated as a complete tritium fuel‐cycle separation system for a fusion power plant. In addition, performance depends on process design, operating conditions, and system integration. Table 1 presents a qualitative comparison of the main hydrogen isotope separation technologies, including their operating principles, temperature ranges, modes of operation, advantages, limitations, and current level of technological maturity.

**TABLE 1:** Comparison of hydrogen isotope separation technologies relevant to fusion tritium fuel cycles, including typical operating temperatures, technology maturity, key advantages, and principal limitations.

Process	Operating temperature^a^	Technology readiness level (Fusion context)	Separation factor [43]	Key advantages	Key limitations/Fusion‐relevant challenges
Cryogenic distillation	20–30 K	Very high (The technology is supported by experience from ITER fuel‐cycle systems and hydrogen isotope separation plants. Operation is well established, and tritium handling has been demonstrated at engineering scale. However, it has not yet been tested as a fully integrated DEMO‐scale system at full tritium throughput).	2.25 @ 24 K	High separation efficiency; proven technology; suitable for large‐scale operation; widely used in fusion fuel‐cycle studies.	Cryogenic operation; high capital and energy requirements; significant tritium inventory; complex system operation.
Palladium‐based membranes (Pd, Pd‐alloys)	473–773 K	High (Proven for hydrogen purification, but experience under long‐term tritium service is limited. Material degradation, including embrittlement, poisoning, and radiation‐induced effects, has not been fully assessed under tritium exposure. Application in integrated fusion fuel‐cycle systems has yet to be demonstrated).	2.0 @ 573 K	High isotopic selectivity; compact, modular design; compatible with catalytic systems and fuel‐cycle processing.	Membrane degradation; high cost; flux reduction over time; limited long‐term tritium operating data; sensitivity to impurities.
Gas chromatography	77–473 K	Moderate (Isotope separation/analytical‐scale purification, with established use in hydrogen isotope analysis. Primarily suited to low‐throughput or batch‐scale applications)	∼2.0 @ 298 K	High separation resolution and analytical precision; suitable for small‐scale purification.	Low throughput; limited scalability; complex operation in continuous systems.
Thermal cycling absorption process	273–473 K	Moderate (Pilot‐scale hydrogen isotope separation demonstrated using metal hydrides. Considered promising for fusion fuel processing loops but still lacks long‐duration tritium operation data and full system integration).	∼2.0 @ 298 K	Simple process; effective isotope separation; lower inventory to cryogenic distillation.	Limited throughput; complex heat management; moderate tritium inventory.
Temperature‐Swing Adsorption (TSA)	20–120 K (Cryogenic adsorption systems)	Low (Mainly studied at bench scale; no tritium‐loop demonstration; limited understanding of tritium adsorption behaviour, thermal cycling performance, and scale‐up).	1.2–55	High isotope selectivity in tailored adsorbents; potential for low tritium inventory.	Cryogenic operation; limited scale‐up.
Pressure‐swing adsorption (PSA)	20–120 K (Cryogenic isotope PSA)	Low (Early‐stage technology for isotope separation by quantum‐sieving PSA; no tritium system demonstration; limited data on adsorbent selectivity and durability).	1.2–55	Fast cycle operation; potential for scale‐up with suitable adsorbents.	Requires highly selective sorbents; not yet demonstrated in fusion‐relevant gas mixtures.

^a^ Based on published studies rather than operational fusion fuel‐cycle systems.

### 4.1 Cryogenic Distillation

Cryogenic distillation for hydrogen isotope separation and tritium fuel enrichment has a rich history in close relation with nuclear technology and fusion research. Its extensive utilisation can be traced back to the nuclear programs, where the demand for isotopic separation spurred advancements in cryogenic technology. Cryogenic distillation utilizes differences in boiling points between hydrogen isotopes (H_2_ (20.4 K), D_2_ (23.5 K) and T_2_ (25.0 K)) [59] to achieve separation. The separation factor can be calculated from the relative vapour pressure of the hydrogen isotopes at a given temperature.

The distillation column contains packing materials which provide a large surface area for vapor‐liquid interaction and promote efficient mass transfer across the column. Through successive condensation and evaporation, the liquid becomes enriched with heavy isotopes.

Cryogenic distillation can achieve very high enrichment and purification of hydrogen isotopes since a distillation column can contain many individual separation stages. Multiple columns are often used with equilibrators in between to convert heteromolecules (HD, DT & HT) which tend to build up in the columns and reduce separation efficiency. The major disadvantage of cryogenic distillation is the large tritium inventory from the liquefied hydrogen isotopes. Additionally, refrigeration (normally using helium refrigeration) is required, and this can be highly energy intensive at the extreme temperatures. During start‐up, the column must be cooled to these extreme temperatures which can take multiple days. If there is a power interruption, the column will rapidly begin to warm and the hydrogen isotopes will boil off. This can cause very high pressures and loss of containment if not mitigated which is a significant safety concern.

The packing material dictates the liquid‐vapour contact and so the separation efficiency. The more efficient the packing, the smaller the column can be made, reducing the tritium inventory in the column. Given the large tritium inventory, this is an important area of research for cryogenic distillation. There are only a few options for packing materials such as Dixon rings or Heli‐pack which are commonly used for other liquid separations in industry [60]. Structured packing has been shown to improve efficiency for other separations [61, 62, 63, 64], so an opportunity exists to improve column packing materials to increase efficiency.

Cryogenic distillation has many applications due to its moderate separation factor and number of separation stages that can be included in a single column. It is possible to achieve high enrichment levels for tritium removal or produce multiple streams of high purity isotopes. However, the high tritium inventory means that it is unsuitable for treating large throughputs of hydrogen isotopes as this would lead to an excessively large column. Hybrid designs incorporating sorbent‐based methods could address these limitations, offering both flexibility and enhanced separation performance. Exploring alternative sorbent‐based hydrogen isotope separation methods offers promising pathways for more energy‐efficient and cost‐effective solutions compared to solely cryogenic approaches. Integrating these sorbent technologies with cryogenic distillation could reduce overall tritium inventories and enhance fusion fuel cycle efficiency by distributing the separation workload across complementary processes [65].

### 4.2 Membranes

The two types of membrane for hydrogen isotope separation are porous membranes and metallic membranes (Figure 4). Porous membranes such as zeolites or MOFs have small pores which demonstrate selective diffusion (Figure 4a). For a non‐selective membrane, the thermal diffusion rate of a gas depends on the square root of the molecular mass. Hence for D_2_ and T_2_, a separation factor of 1.22 should be expected which is very low. At low temperatures, some microporous adsorbents can demonstrate kinetic quantum sieving and chemical affinity‐based separation [66].

**FIGURE 4:**
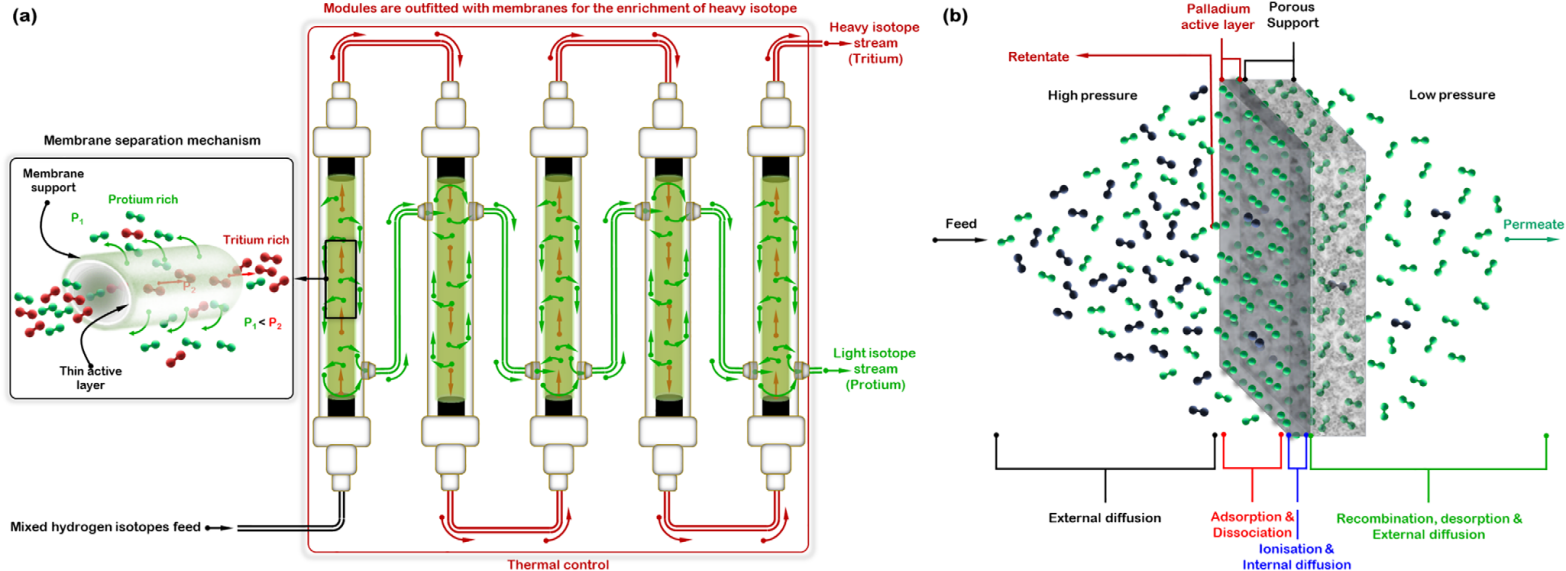
Membrane based separation: (a) Schematic of a membrane‐based process for the preferential removal of protium and the enrichment of deuterium or tritium using multiple membrane modules arranged in series. (b) Schematic illustration of hydrogen isotope transport through a palladium‐based membrane via the solution‐diffusion mechanism, showing molecular adsorption, dissociation, atomic diffusion through the metal lattice, recombination, and desorption.

The other membrane type is metallic membranes which tend to use Pd alloy, as shown in Figure 4b. The hydrogen isotopes pass through the membrane by dissociating at the surface before dissolving into the solid metal. Hydrogen is the only gas that can pass through Pd membranes, and so it is also effective at producing highly pure hydrogen which is its main use outside of fusion. Presently, palladium membranes are used in the fusion fuel cycle to separate impurities, such as helium and other non‐hydrogen gases, from hydrogen isotopologues. The opportunity exists to use palladium permeators as an isotope separation step due to the selective permeation occurring through palladium for lighter isotopes of hydrogen. Higher temperatures (300–500°C) lead to fast permeation rates suitable for hydrogen purification but lower temperatures may be more suited to hydrogen isotope separation due to the higher separation factor [43, 67]. The permeation rate is calculated as the product of the solubility and diffusion coefficient. Conversely despite the higher diffusion coefficient for heavy isotopes, lighter isotopes (protium), have the highest permeation rate due to their higher solubility.

Palladium‐based membranes are highly sought after for their exceptional hydrogen selectivity. However, using pure palladium can lead to issues such as poisoning and rapid mechanical degradation due to embrittlement. Incorporating metals such as Ag [68, 69, 70], Au [71, 72, 73],, Pt [74, 75, 76], Ni [77], Cu [78], Ru, and others into palladium can effectively address these challenges while also enhancing its selectivity [79, 80, 81, 82]. This alloying process not only improves the mechanical strength of palladium‐based membranes but also preserves their high hydrogen permeability.

In addition to palladium, nickel and its alloys also demonstrate excellent permeability for hydrogen isotopes [83, 84]. Nickel‐based alloys, particularly those incorporating copper or iron, can be optimized to enhance both selectivity and durability [85]. Zirconium [86, 87] and perovskite‐based ceramics [88, 89, 90, 91, 92], offer excellent thermal stability and chemical resistance and can be tailored to achieve high selectivity for isotopes like deuterium and tritium [91, 92].

Metals, alloys, and ceramics are among the most promising candidates due to their favourable properties, such as high hydrogen permeability, for separating hydrogen and its isotopes [68, 69, 83, 84, 93, 94, 95, 96, 97, 98, 99, 100, 101, 102, 103, 104, 105, 106, 107, 108, 109, 110, 111, 112, 113, 114, 115, 116, 117, 118, 119]. The potential issue with implementing membranes in the isotope separation system is the pressure drop and capability of the membrane over the broad pressure range that the fuel cycle could require. At low pressures palladium will adsorb hydrogen but it might not leave the material. Another challenge is that to achieve useful enrichment, several membrane stages in series are required which can add significant complexity and difficulty. These membrane stacks require many sealing joints which can fail especially in the elevated temperatures required for Pd alloy membranes and this be difficult to maintain [43, 107]. Achieving optimal permeate and retentate flows for each membrane stage is crucial for efficient operation but can be difficult to achieve for multiple stack arrangements.

### 4.3 Gas Chromatography

In the JET fuel cycle, hydrogen isotopes are separated in batch using frontal displacement chromatography (Figure 5a) [120, 121, 122, 123, 124, 125, 126, 127, 128]. The separation of tritium and deuterium using displacement chromatography relies on their differential adsorption onto a metal hydride forming material within a column. In this process, a flow of D‐T gas is introduced into the column which absorbs to the column packing. Protium is injected as a carrier gas moving through the metal hydride where it selectively absorbs and gradually displaces D_2_ and T_2_. As the tritium is displaced from the solid phase by protium and deuterium a concentration gradient form along the column, where T_2_ is progressively pushed toward the exit, allowing sequential elution and collection of purified T_2_ and D_2_. The Figure 5a illustrates the separation mechanism and gradient formation, highlighting the effectiveness of displacement chromatography [43, 129, 130] in isolating hydrogen isotopes for fusion fuel enrichment.

**FIGURE 5:**
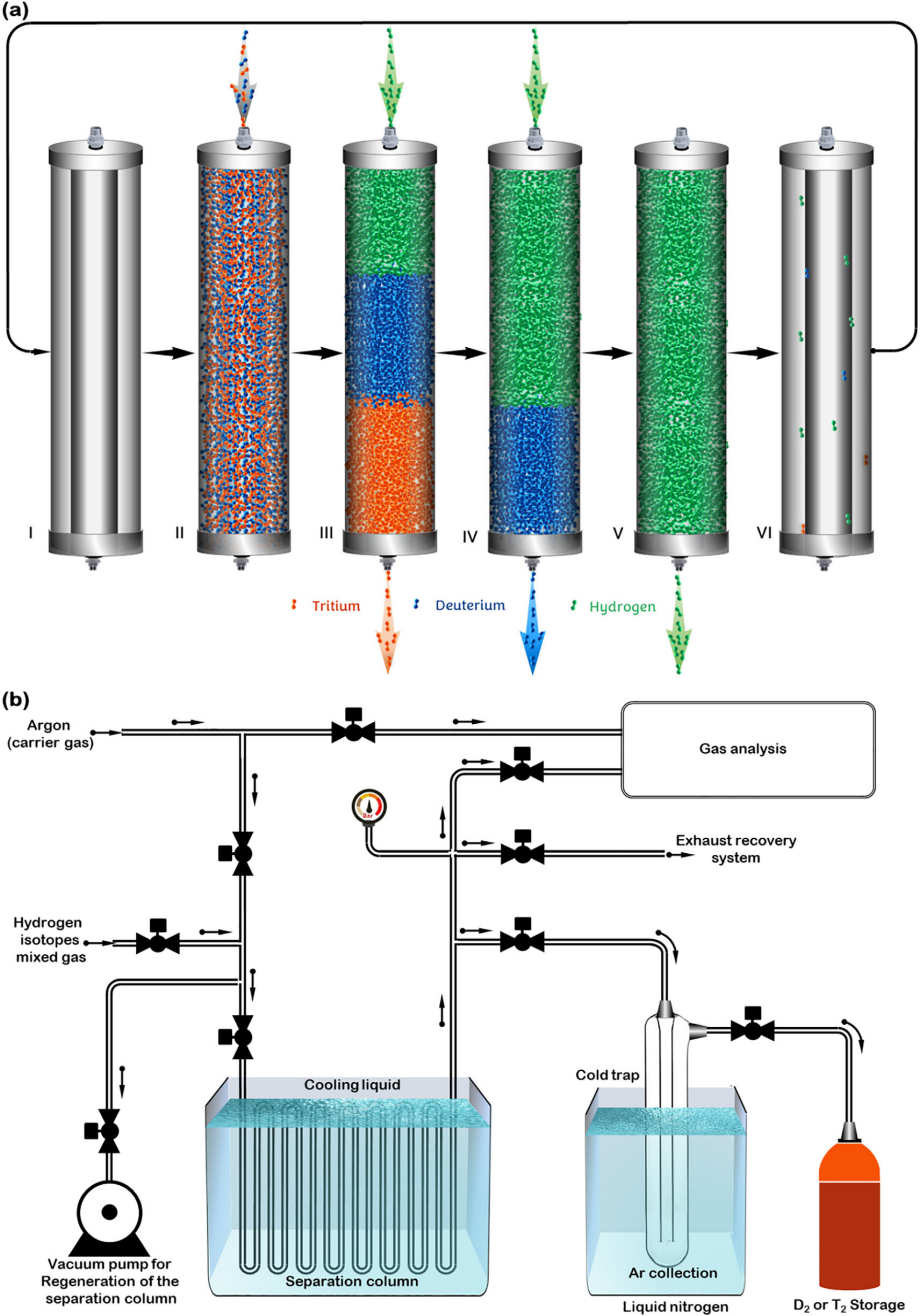
Chromatographic separation: (a) Schematic illustration of the displacement chromatography cycle for the separation of tritium and deuterium using a palladium or palladium‐alloy column, showing adsorption, isotope displacement, product collection, column regeneration, and cyclic operation [43, 129]. (b) Schematic of a cryogenic gas chromatography system for hydrogen isotope separation [38].

The usual sorbent used in displacement chromatography is palladium on a support such such as Pd@Al_2_O_3_ [131, 132] and Pd@kieselguhr [133, 134, 135]. Palladium can separate hydrogen isotopes at room temperature which is a significant advantage compared to alternative processes such as cryogenic distillation. It has a very high uptake for hydrogen (up to an atomic ratio of PdH_0.7_) and high selectivity for lighter isotopes (protium) driven by the difference in enthalpy of hydride formation. Note that almost all other metal hydrides such as Vanadium exhibit the inverse in selectivity, a preference for heavy isotopes. This is because the enthalpy of hydride is much smaller, and so the selectivity in these alternative metal hydrides is driven by the instability of the metal lattice incorporating hydrogen isotopes.

However, Pd is expensive, and to regenerate it requires heating to high temperatures (200°C). This can be slow, which reduces the time availability of the Pd for use in separation.

Cryogenic gas chromatography (CGC) (Figure 5b) has emerged as a promising technique for hydrogen isotope separation due to its high resolution, efficiency, and ability to operate at low temperatures. Zeolites and other microporous adsorbents can preferentially capture heavier isotopes over lighter ones at cryogenic temperatures (77 K), due a to quantum sieving effect (see Section 5). By leveraging the differences in adsorption and diffusion behaviours of hydrogen isotopes under cryogenic conditions, this method offers potential for achieving precise and effective separation of the different hydrogen isotopologues. The advancements in CGC, including improved column materials, optimised temperature control, and enhanced detection systems, are paving the way for improved selectivity and throughput [136]. Further developments such as integrating novel adsorbent materials, optimising operational parameters, and transitioning to a continuous separation process could significantly enhance performance, making CGC a viable solution for large‐scale hydrogen isotope separation and fusion fuel enrichment [130, 137].

### 4.4 Thermal Cycling Absorption Process

Thermal cycling absorption process is a hybrid technique that adapts the key elements of displacement chromatography into a semi‐continuous process. Savannah River National Laboratory, the inventors of TCAP, have been using this technology to separate and purify tritium for over three decades. The UK's Spherical Tokamak for Energy Production (STEP) will also adopt TCAP, supported by a cryogenic distillation system. In this setup, TCAP will handle high‐concentration tritium gas, efficiently separating protium from the stream, while cryogenic distillation will recover trace amounts of tritium from the protium‐rich gas exiting the TCAP system [65].

The TCAP [138, 139, 140, 141, 142] (Figure 6a) uses a separation column filled with packing such as Pd on kieselguhr, and a separate passive column (plug flow reverser) filled with inert packing material. Palladium is deposited onto a porous kieselguhr support for stability (Pd/K) [134, 139, 143]. The passive column contains light isotopes which are passed into the column displacing the heavy isotopes, similarly to displacement chromatography. After each cycle, the column is heated and the flow is reversed enabling the light isotopes to be produced at the top of the column, some of which is diverted to be stored in the passive column for future cycles. At higher temperatures, the selectivity and uptake of the packing is lower which preserves the concentration profile despite the reverse in flow (Figure 6a). After several cycles, the system's preference for lighter isotopes forms a concentration gradient, separating isotopes to opposite ends of the column.

**FIGURE 6:**
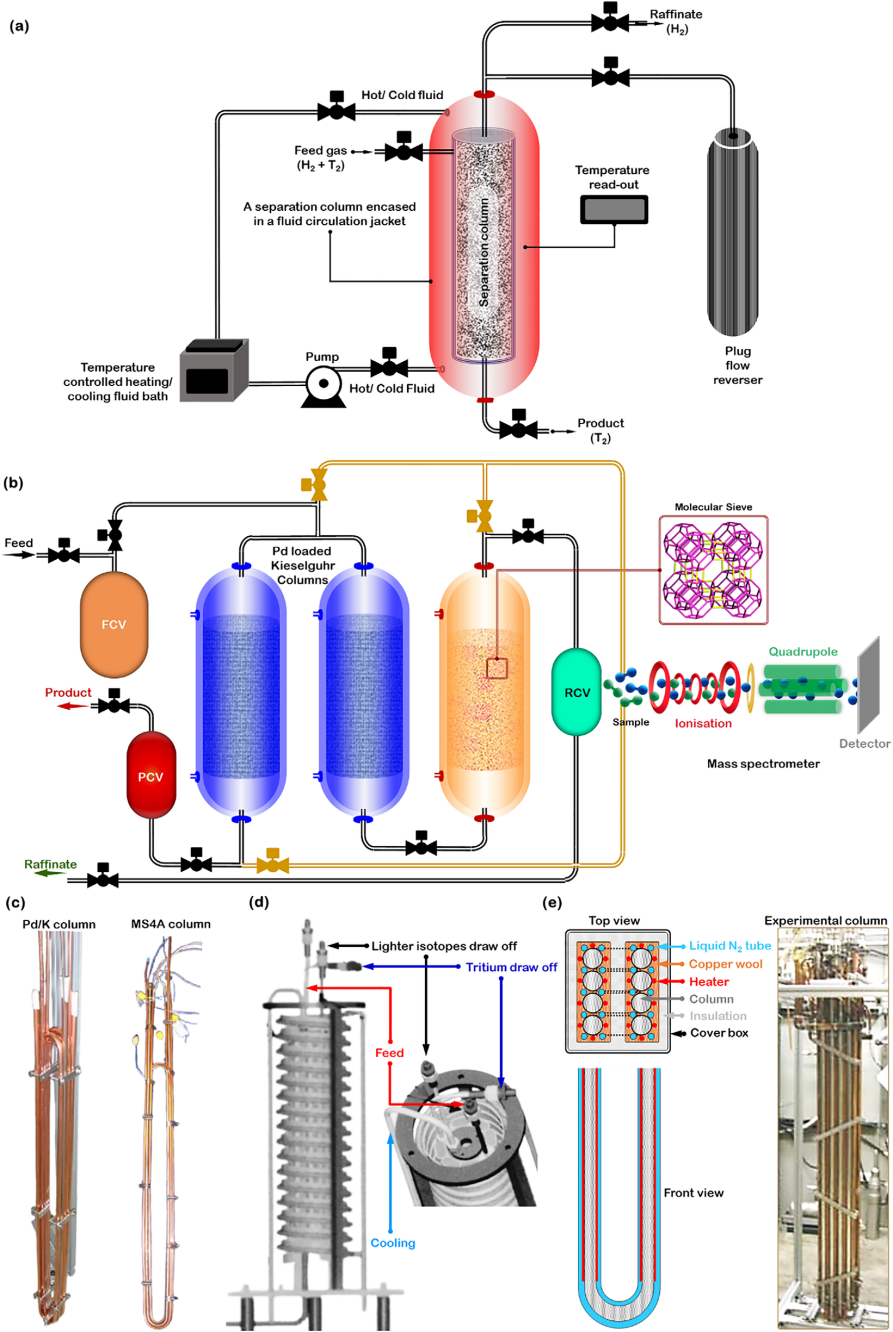
Temperature controlled adsorption processes: (a) Schematic of the thermal cycling absorption process (TCAP) for hydrogen isotope separation using a Pd/K column and plug‐flow reverser [43]. (b) Schematic representation of a TCAP system incorporating a Pd/K absorption column and an inverse column packed with molecular sieves, including in situ residual gas analyser. (c) Representative images of Pd/K and molecular sieve columns. Adopted with permission [143]. Copyright 2011, Taylor & Francis Group. (d) Schematic and experimental setup of the CEA/Valduc TCAP device for hydrogen isotope separation. Adopted with permission [142]. Copyright 2001, Elsevier Science B.V. (e) Compact TCAP column design and corresponding experimental image. Adopted with permission [141]. Copyright 2008, Taylor & Francis Group.

Heating and cooling of the column is required for each TCAP cycle which can be slow and so reduce the time available for isotope separation. TCAP systems can utilise various heating and cooling systems, including direct electrical heating, hot gas or steam injection, and heat exchangers for heating, while cooling methods include cold gas (or liquid nitrogen) injection, and air cooling to achieve efficient gas separation.

TCAP configurations have been further refined, replacing the PFR with molecular sieves in an inverse column setup [120, 143]. Further advancements in TCAP technology have focused on enhancing energy efficiency through improved heating and cooling mechanisms. Given the extensive research already available on TCAP's operational improvements, this review will not cover its development in detail [139, 140].

Thus Figure 6b–e showcases an improved TCAP design incorporating an inverse zeolite column to boost the separation efficiency. In the conventional TCAP setup (as depicted in Figure 6a), the system utilizes a Pd/K column alongside a PFR. However, the PFR is largely passive, serving only as a space for the Pd/K column to regenerate, without actively contributing to isotope separation. This configuration is effective at producing high‐purity heavy isotopes (the product) but struggles to achieve high‐purity light isotopes (the raffinate). To address this limitation, the concept of replacing the passive PFR with an active inverse column was developed. The inverse column not only provides the regeneration function but also introduces an isotopic effect opposite to that of the Pd/K column. Molecular sieves were chosen for the inverse column due to their ability to adsorb significant amounts of hydrogen at liquid nitrogen temperatures. Molecular sieves demonstrate different equilibrium pressures for hydrogen isotopes, which effectively reverses the separation behaviour of the Pd/K column. This complementary action between the two columns enables efficient separation of both light and heavy hydrogen isotopes, thereby enhancing the overall performance of the TCAP system.

A key gap in the literature is the lack of focus on the sorbent materials used within the active and inverse columns. Despite TCAP's advancements over the last four decades, the primary materials reported are still limited to Pd/K and zeolite 4A. This highlights a significant opportunity for further exploration into alternative adsorbents and absorbents that could optimize TCAP performance.

### 4.5 Temperature Swing Adsorption and Pressure Swing Adsorption

Adsorbents have significant promise for hydrogen isotope separation. Hydrogen isotopes display only minor differences in physical and chemical properties and so can be difficult to separate at room temperature using adsorbents. However, at cryogenic temperatures (<77 K), when confined in the pores of adsorbents, hydrogen isotopes demonstrate differences in their quantum properties. This effect has been termed quantum sieving and can achieve very high isotope selectivity. As such adsorbents have significant potential to be used for hydrogen isotope separation but have not yet been tested at large scales in adsorption columns. At this developmental stage it is important to consider which adsorption cycle is most suitable. Even if adsorbent shows promise for hydrogen isotope separation, it may not be practical or efficient when operated within a large‐scale adsorption cycle.

The two most common adsorbent processes for gas separation are TSA or PSA. TSA leverages temperature changes to modulate the adsorption and desorption of gases on the surface of selected adsorbents [144, 145, 146, 147, 148]. At low temperature the gas mixture is fed into an adsorbent column which selectively absorbs one of the components. The residual non‐adsorbing gases are collected at the end of the column. Once the adsorbent is fully saturated it cannot adsorb anymore gas and so it must be regenerated. The column is heated to higher temperature which drives the desorption of the gases from the adsorbent, allowing the adsorbing gases to be collected, before the cycle is completed. Multiple columns are often used so that one column can be adsorbing while the another is being regenerated, enabling processing of a continuous throughput of gas. TSA is a well‐established technology for gas separation in various industrial applications involving gaseous mixtures. However, in the context of fusion science and hydrogen isotope separation, a pure TSA process is not currently being developed. Temperature changes in adsorbent columns can be very slow which reduces the available time for adsorption and so the throughput of the system. However, temperature changes provide a strong driving force for adsorption/desorption. Hence TSA is used when the adsorbent used for separation has a high enthalpy of adsorption. This is the case for many adsorbents tested in the literature such as MOFs and zeolites which rely on chemical affinity quantum sieving and so have high heats of adsorption.

In contrast to TSA, the PSA process relies on a change in pressure to initiate adsorption and desorption. The column is initially pressurised to atmospheric pressure or above, this drives adsorption. To regenerate the adsorbent, the column pressure is reduced to low gas pressure or vacuum pressure which drives desorption. PSA is well suited to large scale gas separation because pressure‐changes can be completed much faster than temperature changes, even in very large columns (Figure 7a). It also offers significant energy savings compared to thermal‐based processes, because pressure changes require much less energy than equivalent thermal changes. As a result, PSA is widely adopted in industrial gas purification applications such as hydrogen purification [127, 149, 150, 151, 152, 153, 154, 155, 156, 157, 158, 159]. The drawback of PSA is that pressure‐swing operation provides a weak driving force for adsorption/desorption. This has two significant implications for the application to hydrogen isotope separation. Firstly, this limits the application of PSA to adsorbents systems with a weak adsorption affinity to hydrogen. Secondly even with a suitable adsorbent, pressure‐swing will not fully regenerate the adsorbent which reduces its working capacity and tends to reduce the selectivity. Hence one of the primary challenges in PSA based hydrogen isotope separation is identifying an optimal sorbent.

**FIGURE 7:**
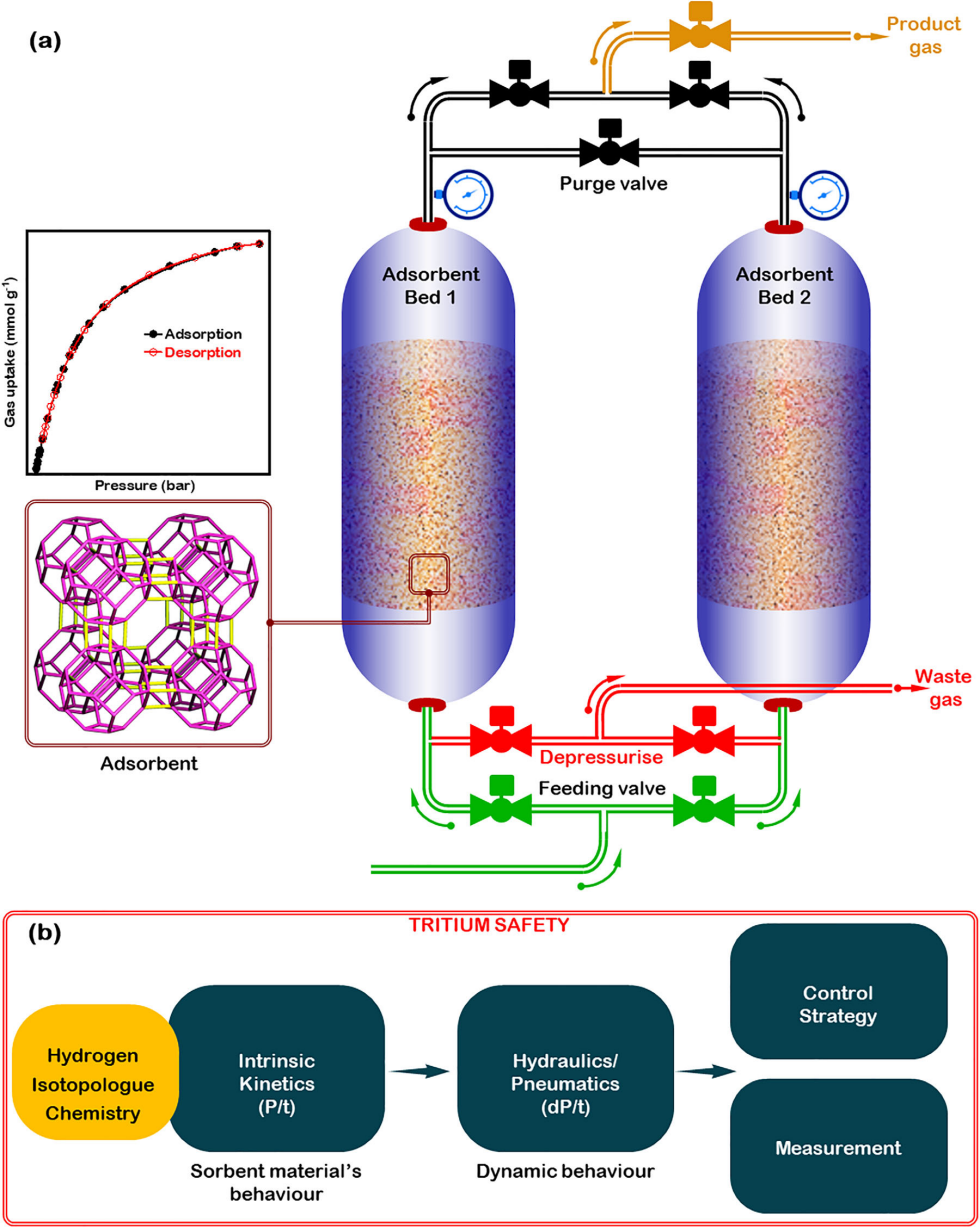
(a) Schematic of a conventional two‐bed PSA system. (b) Key considerations for the design of sorbent‐based hydrogen isotope separation systems. The red boundary highlights challenge specific to tritium fuel cycle operation in D‐T systems.

PSA for hydrogen isotope separation remains primarily in the developmental stage. Despite its developmental status, PSA has been explored for isotope separation for fusion powerplants. One of the few studies to explore the performance of PSA for hydrogen isotope separation is by Kotoh et al. who tested commercially available zeolite 5A and 13X at 77 K [150, 152, 153, 154, 155, 156, 157, 158, 160, 161, 162, 163]. It was found these adsorbents had reduced separation performance when compared to the equilibrium adsorption isotherms, because the vacuum pressures used to regenerate the adsorbent in pressure‐swing operation were unable to fully desorb the D_2_ from the zeolite. From the D_2_ adsorption isotherm of zeolite 5A at 77 K, it would seem apparent that a very low vacuum pressure should be able to remove all the adsorbed gas. However, in a real scale adsorption column, the actual desorption pressure is limited not only by the vacuum pump, but the additional gas pressure needed to overcome resistances to flow from the packed bed and diffusion in the adsorbent pores. Knowing the operating pressures and using the isotherm data, the working capacity and selectivity of an adsorbent in pressure‐swing operation can be calculated.

Effective hydrogen isotope separation using PSA will require adsorbents with high selectivity, reversible adsorption behaviour, and sufficient capacity. TSA may be more suitable to adsorbents with high heat of adsorption for hydrogen, since pressure‐swing operation becomes less effective with these adsorbents. Ongoing research into advanced materials continues to expand the range of candidate sorbents, and those demonstrating strong performance in PSA may also be applicable to other sorbent‐based separation technologies. There is a clear interdependence on process selection and the sorbent properties. The performance of PSA, TSA, and gas chromatography depends on adsorption thermodynamics, isotope selectivity, mass‐transfer kinetics, and sorbent regeneration behaviour. At the same time, each process places specific demands on adsorption capacity, operating conditions, heat management, structural stability, and compatibility with tritium‐containing environments. In fusion applications, factors such as radiation tolerance, isotope‐exchange reactions, and tritium retention must also be considered. The effectiveness of an adsorption‐based separation system therefore depends on both the process design and the properties of the sorbent, and these factors must be evaluated together when selecting suitable materials.

## 5 Sorbent Selection for Isotope Separation Processes

For all sorbent‐based isotope separation technologies the separation performance depends highly on the sorbent in the column. It's important to thoroughly characterise and select appropriate sorbents using thorough data analysis and simulation to understand how to design the isotope separation system. An ideal sorbent for hydrogen isotope separation should possess the following key characteristics:

- Mild operating temperatures—Effective separation at or near room temperature (or at least above 77 K), minimising energy input for thermal control.
- High adsorption capacity
- High selectivity between hydrogen isotopes.
- Fully reversible adsorption–desorption behaviour, allowing consistent performance over many cycles without requiring regeneration under harsh conditions.
- Low tritium retention, avoiding liquid tritium accumulation and reducing radioactive inventory and associated safety concerns.
- Chemical and structural stability ensuring minimal degradation over extended operation, especially important due to the difficulty of maintaining tritium systems.
- Resistance to radiolysis and tritium‐induced damage, which is critical for materials operating in radioactive environments. This is a particular concern for organic compounds, and some sorbents have been tested with radiation exposure (see Section 5.2).
- Scalability and process compatibility, meaning the material should be available or producible at scale and compatible with existing or planned separation systems.

Figure 7b shows factors that must be considered when designing a sorbent‐based separation system for hydrogen isotope separation. Sorption isotherm and Thermal Desorption Spectroscopy (TDS) experiments provide small scale equilibrium data and are an important first step in understanding the potential of an adsorbent and the conditions required for hydrogen isotope separation. Once it has been shown a sorbent shows promising properties, dynamic breakthrough analysis and pilot scale adsorption column testing are required to find the isotope selectivity and adsorption kinetics under more relevant scales for separation processes. The findings will dictate the control philosophy, measurement systems must be able to track at the pace and precision required.

Mass transfer and hydraulics (momentum balance) within a column provide information about process control and column optimisation. For example, Chahbani et al. [164] highlight the critical role of selecting appropriate mass transfer kinetic models for accurately simulating PSA processes, particularly during pressurisation and depressurisation steps when pressure is varied over time. They state that the behaviour of sorbent columns with respect to varying pressure steps is underexplored for fuel cycle PSA systems The findings underscore the necessity of performing experimental campaigns to study the kinetic and hydraulic performance of sorbents for all sorbent‐based separation processes. Table 2 introduces the main classes of adsorbents being considered for hydrogen isotope separation and their specific advantages and disadvantages.

**TABLE 2:** Overview of the major sorbent classes investigated for hydrogen isotope separation and their key characteristics, advantages, and limitations.

Sorbent class	Representative characteristics	Advantages	Disadvantages
Zeolites	Crystalline aluminosilicate frameworks with well‐defined microporous structures [165].	High chemical, thermal, and radiation stability; commercially available; demonstrated compatibility with tritium environments; adsorption properties can be tailored through ion exchange or incorporation of metal cations [166].	Hydrogen isotope selectivity is generally low, and effective separation often requires cryogenic temperatures. Selectivity can be improved through the incorporation of cations or open metal sites.
Porous carbons	Carbonaceous materials (e.g., activated carbons, carbon molecular sieves) with tunable pore structures produced by pyrolysis or activation [167].	High surface area; excellent chemical and thermal stability; scalable synthesis; pore structures suitable for molecular sieving.	Generally low isotope selectivity except at cryogenic temperatures; heterogeneous pore structures make performance difficult to predict; introducing strongly adsorbing sites remains challenging.
Porous framework materials (MOFs, COFs, porous organic cages, and related porous organic materials)	Crystalline or amorphous porous materials with highly tunable pore size, chemistry and functionality through metal nodes, covalent linkages or organic building blocks [168].	Exceptional structural tunability; high surface area; potential for high isotope selectivity through open metal sites, quantum confinement, framework flexibility or tailored pore chemistry.	Variable chemical, thermal, and radiation stability; challenges in large‐scale synthesis; limited data on tritium exposure, including effects of radiolysis and isotope exchange.
Metal hydrides	Metals and intermetallic compounds capable of reversibly absorbing hydrogen isotopes into their crystal lattice.	High isotope selectivity at near‐ambient temperatures; high hydrogen storage capacity enables compact separation systems; well established for hydrogen isotope processing (e.g., TCAP).	Thermal energy required for hydrogen desorption; embrittlement, lattice damage, and powdering may occur during prolonged tritium exposure and cycling [140].

### 5.1 Metal Hydrides for Absorption Processes

A key factor in advancing isotope separation systems lies in the development of materials alongside process optimization. In the case of TCAP systems, only two types of palladium supports (alumina and kieselguhr) have been widely utilised. Recent discoveries regarding the adsorption behaviour of hydrogen isotopes on specific palladium surfaces and facets show enhanced kinetics for certain facets. Using this knowledge, it would be possible to develop more efficient sorbents for TCAP or other chromatographic process.

Palladium and its alloys have been known for its high hydrogen solubility and permeability [69, 94, 96, 97, 98, 99, 100, 101, 102, 106, 107, 112, 113, 114, 115, 169, 170, 171]. It allows hydrogen isotopes to diffuse rapidly through its lattice, with slight variations in permeation rates for different isotopes. Palladium hydride (PdH_x_) forms when hydrogen is adsorbed at the palladium surface and absorbed into the metal lattice, resulting in two distinct face‐centered cubic (fcc) phases. At low hydrogen content (*x* < 0.01), an α‐phase solid solution forms, which fully transforms into an expanded lattice β‐phase interstitial alloy at high hydrogen concentrations (*x* ≈ 0.7). In bulk palladium, these phases coexist at 0.01 < *x* < 0.7. Single‐crystalline palladium nanocrystals undergo abrupt α‐ to β‐phase transitions without phase coexistence [172]. and could allow improved hydrogen isotope separation due to a link between palladium crystal facets and faster hydrogen transport kinetics [143, 173, 174].

Nanometer‐sized materials have gained significant attention due to their unique physical and chemical properties. Single‐particle experiments have shown that the hydrogen absorption rate in cubic palladium nanocrystals is controlled by the formation of a β‐phase nucleation site that propagates through the crystal. Johnson et al. [169], utilize in situ X‐ray diffraction to track both hydrogen absorption and desorption in palladium nanocrystals, which enables the identification of distinct absorption and desorption mechanisms in nanocrystals with exclusively (111) or (100) facets (Figure 8). Nanocrystals with 111 surface facets (Pd_oct_) had slower kinetics than the 100 or mixed facet crystals due to 111 facets having a higher recombination energy barrier. The variations in phase transition rates and behaviour between Pd_mix_, Pd_cube_, and Pd_oct_ highlight how surface structure influences hydrogen dynamics (Figure 8).

**FIGURE 8:**
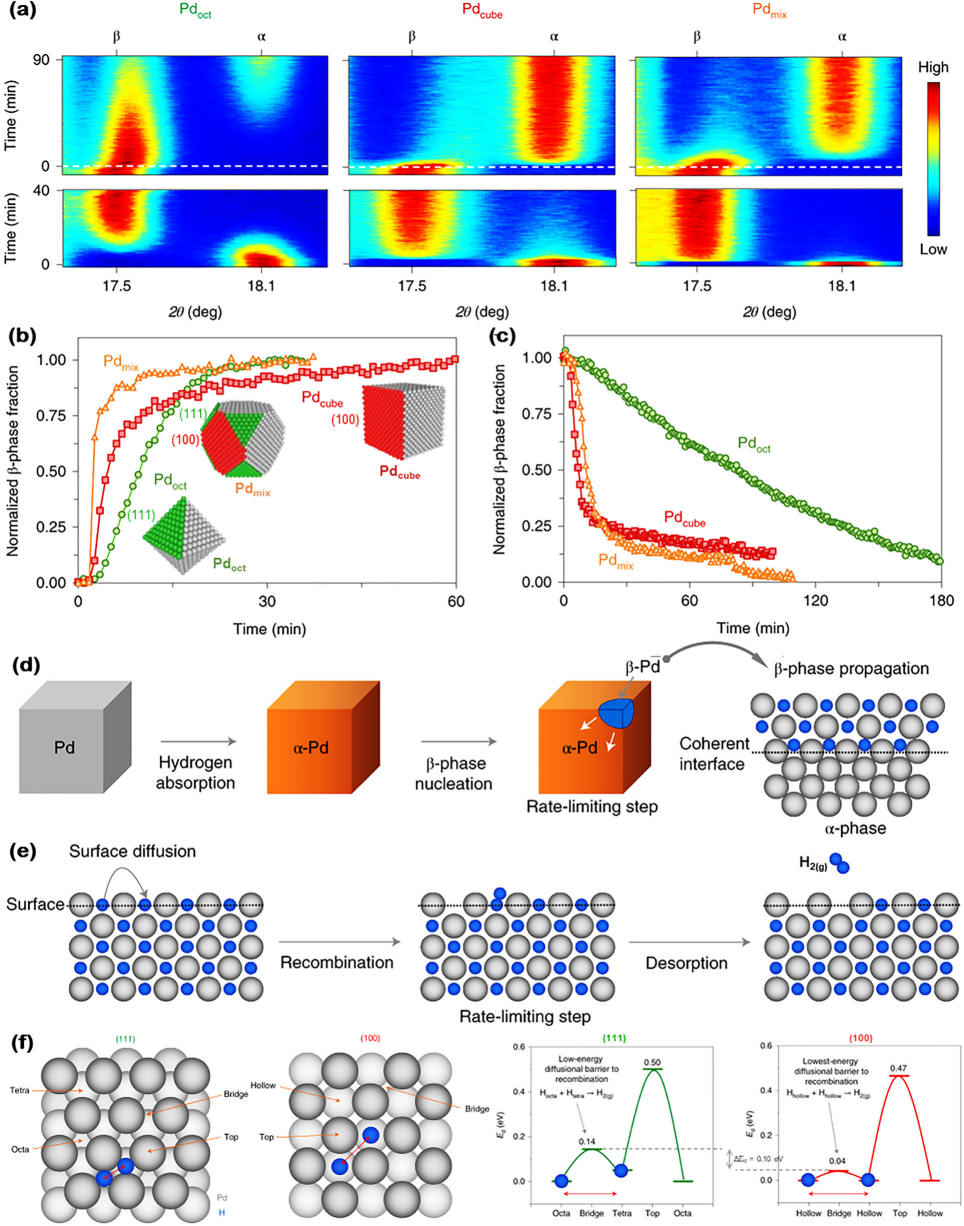
Hydrogen absorption‐desorption behaviour in Pd nanocrystals. (a) Plots for hydrogen absorption and desorption cycles for the octahedral (Pd_oct_), cubic (Pd_cube_), and mixed structure (Pd_mix_) palladium nanocrystals. (b) Normalised β‐phase fraction during hydrogen absorption. (c) β‐phase decay during hydrogen desorption for different nanocrystal morphologies. (d) Schematic of β‐phase nucleation and growth during hydrogen absorption in a Pd nanocrystal. (e) Schematic of surface‐limited hydrogen recombination as the rate‐limiting step during desorption. (f) Hydrogen adsorption sites and relative energetics on Pd (111) and (100) surfaces, highlighting differences in adsorption stability and diffusion barriers. Reproduced with permission [169]. Copyright 2019, Springer Nature Limited.

The rate constants for hydrogen absorption have been shown to increase with the number of vertices in the Pd crystal. Johnson et al.,^154^ found nucleation of the β‐phase occurred at vertices experiencing tensile strain, followed by the coherent expansion of this phase throughout the nanocrystal (Figure 8d) [175, 176]. The rate of hydrogen absorption is constrained by β‐phase nucleation at vertices, while desorption is controlled by the surface‐limited recombination of hydrogen atoms into H_2_. The different low‐index facets can lead to significantly different desorption rates and offers a new approach for managing hydrogen uptake and release.

Ulvestad et al. [175], investigated in situ 3D strain dynamics in single Pd nanocubes during hydriding revealing that in the dilute hydrogen α‐phase, strain was highly particle‐dependent while in the hydrogen‐rich β‐phase, strain behaviour became particle‐independent and driven by elastic effects (Figure 9a–c). Such effects are important in considering how hydriding conditions will affect particle strain and so the longevity of the Pd absorbent particles. Adsorption‐induced strain and phase behaviour can influence selectivity and reversibility.

**FIGURE 9:**
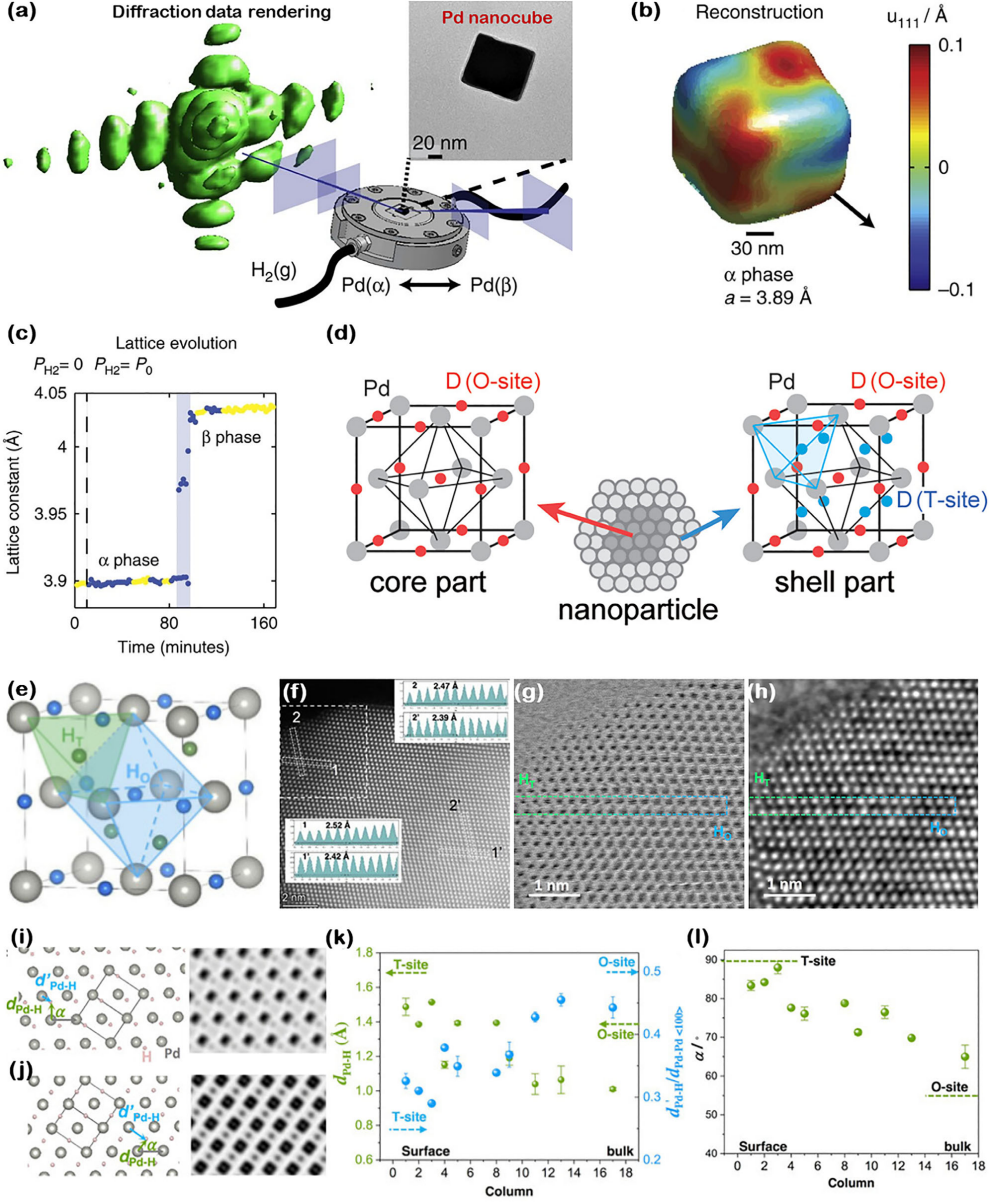
Structural and atomic‐scale characterization of Pd and PdH_x_ systems. (a) Experimental setup and diffraction isosurface reconstruction of an α‐phase Pd nanocube. (b) Reconstructed (111) lattice displacement field from electron density isosurfaces. (c) Time evolution of the lattice constant of a single Pd nanoparticle under increasing H_2_ partial pressure. (a‐c) Reproduced with permission [175]. Copyright 2015, Springer Nature Limited. (d) Schematic of β‐PdH structure showing hydrogen occupancy at tetrahedral (T) and octahedral (O) interstitial sites. Reproduced with permission [170]. Copyright 2016, American Chemical Society. (e) Representation of hydrogen T‐ and O‐site interstices in Pd. (f) HAADF‐STEM image of truncated octahedral Pd nanoparticles with Pd‐Pd spacing analysis. (g,h) dDPC and iDPC‐STEM images of the PdH_x_ nanoparticle corner region. (i,j) Structural models and simulated dDPC images of PdH_x_ along the (110) zone axis for T‐site and O‐site occupancy. (k,l) Quantitative analysis of hydrogen occupancy variation from surface to bulk. (e–l) Reproduced with permission [177] Copyright 2020, WILEY‐VCH GmbH.

Tetrahedral sites T‐sites in Pd nanoparticles are of particular interest (Figure 9d), as they are not typically occupied in bulk Pd or other fcc metal hydrides and can provide unique adsorption environments that can enhance isotope selectivity and diffusion [170]. Lin et al. [177] found that T‐site occupation was enhanced in Pd nanoparticles (Figure 9e–l). They provided direct imaging of subsurface hydrogen atoms absorbed in palladium nanoparticles. Surface occupation of T sites enhances hydrogen isotope separation by amplifying zero‐point energy differences between isotopes, leading to greater selectivity due to stronger isotope‐dependent absorption behaviour in these more confined, surface‐accessible environments.

These studies show that there is significant potential to tailor the kinetics and selectivity of Pd through modification of the surface and inclusion of Pd nanoparticles. One practical issue with using Pd nanoparticles for TCAP and other applications is that their small size will severely limit their hydrogen uptake capacity when compared to bulk Pd or supported Pd films.

### 5.2 Adsorbents

In recent years, adsorbents such as MOFs, zeolites, and covalent organic frameworks (COFs) have become of the most promising materials for the separation of hydrogen isotopes due to their very high selectivity, but have not been tested at large scales. Compatibility of these adsorbents with practical scale separation methods including PSA and TCAP is one of the key concerns because almost all adsorbents tested require extreme cryogenic temperatures (<77 K). Although zeolites have been proven effective in tritium environments, for organic based adsorbents, the tritium compatibility is a concern. Organic compounds are liable to exchange with tritium, which can then radioactively decay and decompose the organic structure of the adsorbent.

#### 5.2.1 Fundamental Gas Separation Principles

As discussed and proved by Hyunchul and Moon in their review and research articles [178, 179, 180, 181, 182], traditional gas separation in porous materials generally involves three primary mechanisms: molecular sieving (size–shape exclusion), kinetic separation (diffusion rates), and thermodynamic equilibrium (adsorbate‐adsorbent interactions).

Separating Hydrogen isotopes which have nearly identical physicochemical properties and molecular size is very challenging using conventional approaches such as molecular sieving in adsorbents. It can be achieved through quantum sieving and through chemical affinity. The former leverages quantum effects in kinetic or thermodynamic equilibrium separation, and the latter leverages strong adsorbate‐sorbent interactions.

Beenakker et al. [183] proposed a new approach known as quantum sieving which only occurs at very low temperatures. When molecules are confined in pores of similar size to the molecules, the gap size between the molecule and pore wall becomes so small that at low temperatures it is similar in scale to the de Broglie wavelength of the molecule. Since the de Broglie wavelength is inversely proportional to the particle mass, this effect is very sensitive to molecular mass (deuterium is twice as heavy as protium). Protium being the lightest is most affected by quantum sieving and has the highest zero‐point energy (ZPE) (Figure 10a–c) [178, 183]. The additional energy term from the ZPE makes it more difficult for protium to adsorb compared to deuterium or tritium. Hence quantum sieving leads to a selectivity for heavy hydrogen isotopes adsorption which is the inverse selectivity compared to palladium.

**FIGURE 10:**
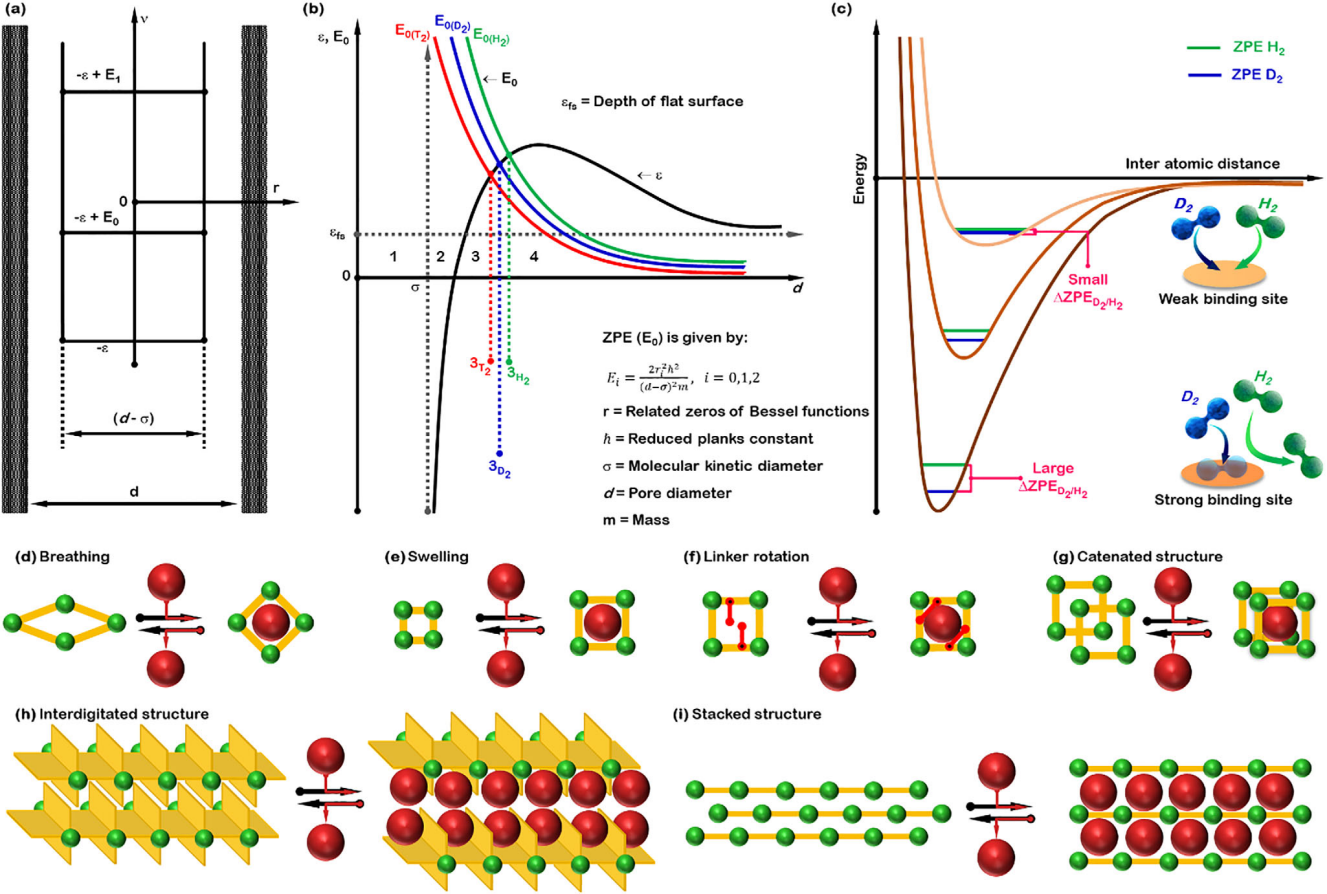
(a) Schematic representation of molecular potential energy (ν) and transverse energy levels (Ei) in a confined channel with d‐σ∼λ. (b) Variation of well depth (*ε*) and zero‐point energy (*E*_0_) of isotopes as a function of pore diameter, highlighting regimes of classical transport and quantum sieving (Region 3). (c) Schematic illustration of differences in potential well depth corresponding to isotope‐framework interaction strength [178]. (a–c) Adopted with permission [178]. Copyright 2020 WILEY‐VCH Verlag GmbH & Co. KGaA, Weinheim. (d–i) Representative structural responses of flexible MOFs, including breathing, swelling, linker rotation, catenation, interdigitation, and stacking. Adopted with permission [184]. Copyright 2021 The Royal Society of Chemistry and the Chinese Chemical Society.

Quantum sieving can occur due to the confinement of narrow pores and is known as Kinetic Quantum Sieving (KQS). Quantum sieving can also occur due to the confinement from high affinity adsorption sites such as open metal sites in MOF adsorbents. This mechanism, known as Chemical Affinity Quantum Sieving (CAQS), leads to enhanced selectivity and allows isotope separation at elevated temperatures due to deeper adsorption potentials. KQS requires temperatures of 77 K or below and can deliver impressive separation factors approaching the boiling temperature for hydrogen (25 K). There is little energy efficiency benefit to designing isotope separation systems that need to operate at such low temperatures, and cryo‐distillation may be much more effective. Using the CAQS effect allows for much more amenable separation temperatures > 77 K. A comparison of adsorbents tested for hydrogen isotope separation is shown in Figure 11.

**FIGURE 11:**
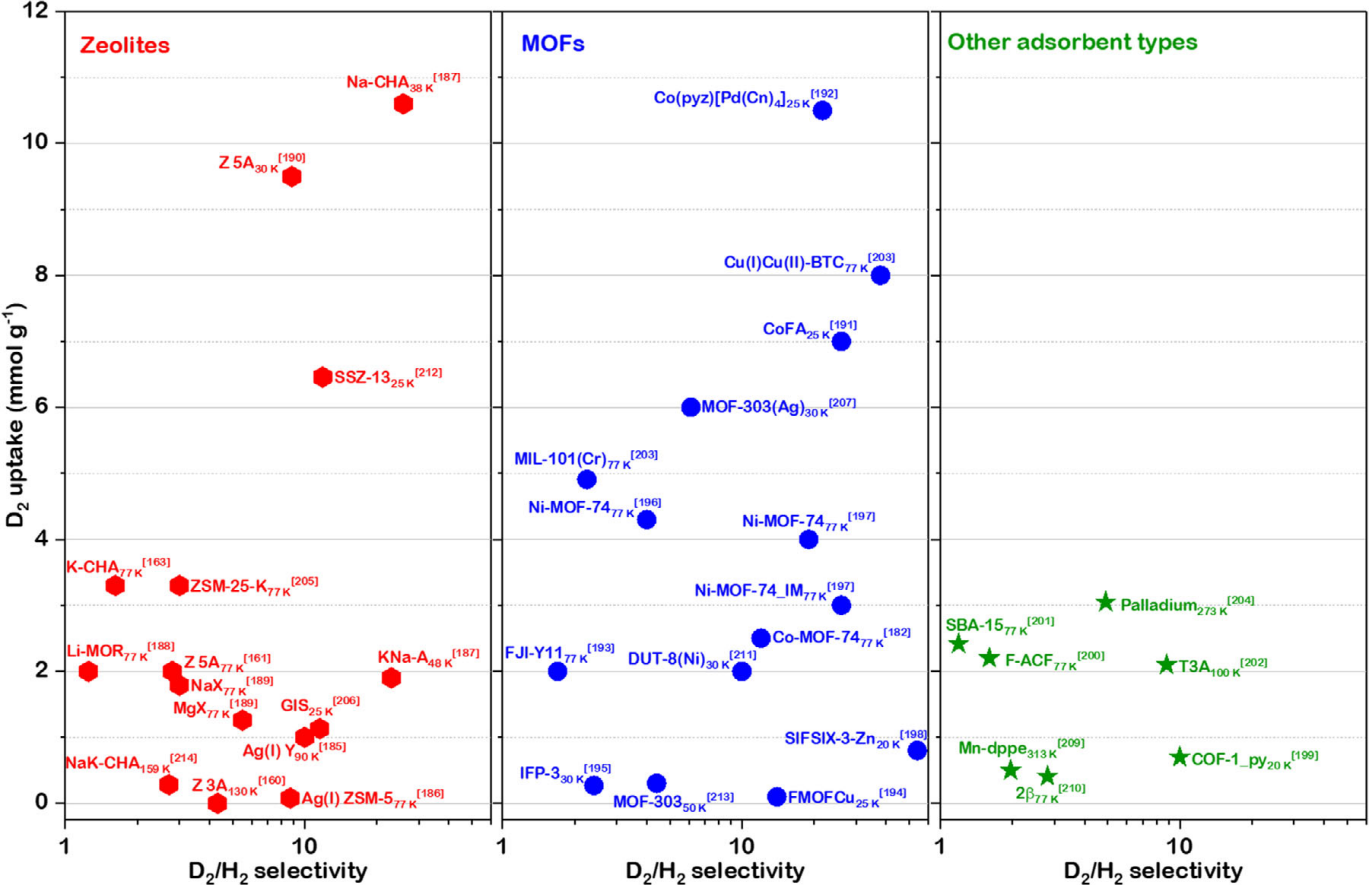
Selectivity and adsorption uptake of adsorbents reported in the literature [160, 161, 163, 182, 185, 186, 187, 188, 189, 190, 191, 192, 193, 194, 195, 196, 197, 198, 199, 200, 201, 202, 203, 204, 205, 206, 207, 208, 209, 210, 211, 212, 213, 214]. Palladium_273 K_—for purpose of comparison to adsorbents, separation factor value of H_2_/D_2_ is inverse and derived as the product of H_2_/HD and HD/D_2._

#### 5.2.2 MOFs: Enhancing Hydrogen Isotope Separation Efficiency

MOFs are crystalline porous structures composed of metal nodes connected by organic linkers. MOFs are extremely versatile and can have a wide range of pore sizes and shape which can contain open metal sites with a strong affinity for adsorbing gaseous hydrogen isotopes. Their dynamic characteristics, such as breathing, swelling, linker rotation, and substructural displacement (Figure 10d–i), allow them to adapt their pore dimensions [184, 215, 216, 217]. These transformations enhance mechanisms like KQS and CAQS, enabling MOFs to exploit subtle differences in the mass and size of hydrogen isotopes effectively. FitzGerald and colleagues [196] were among the pioneers in exploring CAQS within the MOF‐74 series (Fe, Co, Ni) for hydrogen isotope separation under low‐pressure conditions across a temperature range of 77–150 K. Their findings highlighted that Ni‐MOF‐74 exhibited a higher isosteric heat of adsorption for D_2_ compared to H_2_, driven by ZPE differences. This resulted in a notable D_2_/H_2_ selectivity of 5.0 at 77 K, with the highest selectivity of 11.8 achieved at 60 K and 30 mbar.

MOFs containing open metal sites not only have high hydrogen isotope selectivity but can operate at more mild temperatures. Recently Kitayama et al. [209] reported unprecedented room temperature hydrogen isotope separation using an unsaturated organometallic complex with an exceptionally high heat of adsorption for H_2_ of ‐50.2 kJ/mol. The adsorbent was able to achieve D_2_/H_2_ of 1.98 at 313 K as calculated using IAST. Hofmann‐type MOFs have been tested by Ha et al. [192] and have exceptionally high density of open metal sites. This leads to a high D_2_/H_2_ selectivity of 21.7 and very high D_2_ uptake of 10 mmol/g at 25 K for Co(pyz)[Pd(CN)_4_]. Chen et al. [218] tested a mixed zinc/copper MOF (M′MOF) material demonstrating exceptional hydrogen isotope separation performance due to its microporous structure and open copper metal sites.

As illustrated in Figure 12, the synergistic interplay between CAQS and KQS has been effectively utilized in several MOFs materials, including MOF‐74‐IM (Figure 12a) [197], MOF‐303 (Figure 12b) [213], UiO‐67_Be (Figure 12c) [219], and Cu(I)Cu(II)‐BTC (Figure 12d) [203], to achieve remarkable hydrogen isotope separation performance. Kim et al. [197] incorporating imidazole molecules into MOF‐74 to form MOF‐74‐IM restricted the pores. MOF‐74 contains open metal sites which led to a high selectivity through the CAQS effect of 17 at 77 K which was enhanced to 26 due to the pore restriction by the IM molecules. Dong et al. [219] investigated doping UiO‐67 with Be, Mg or Ca using a computer model which predicted Be‐doped sites would have enhanced D_2_/H_2_ selectivity at 77 K (Figure 12c). Hu et al. [203] reported a synthetic HKUST‐1‐derived microporous mixed‐valence Cu(I)Cu(II)‐BTC. The researchers were able to replace some of the Cu(II) open metal sites in HKUST‐1 for Cu(I) sites which have much higher affinity for hydrogen leading to enhanced D_2_/H_2_ isotope separation (Figure 12d). This material achieved an impressive D_2_/H_2_ selectivity of 37.9 at 30 K, outperforming HKUST‐1.

**FIGURE 12:**
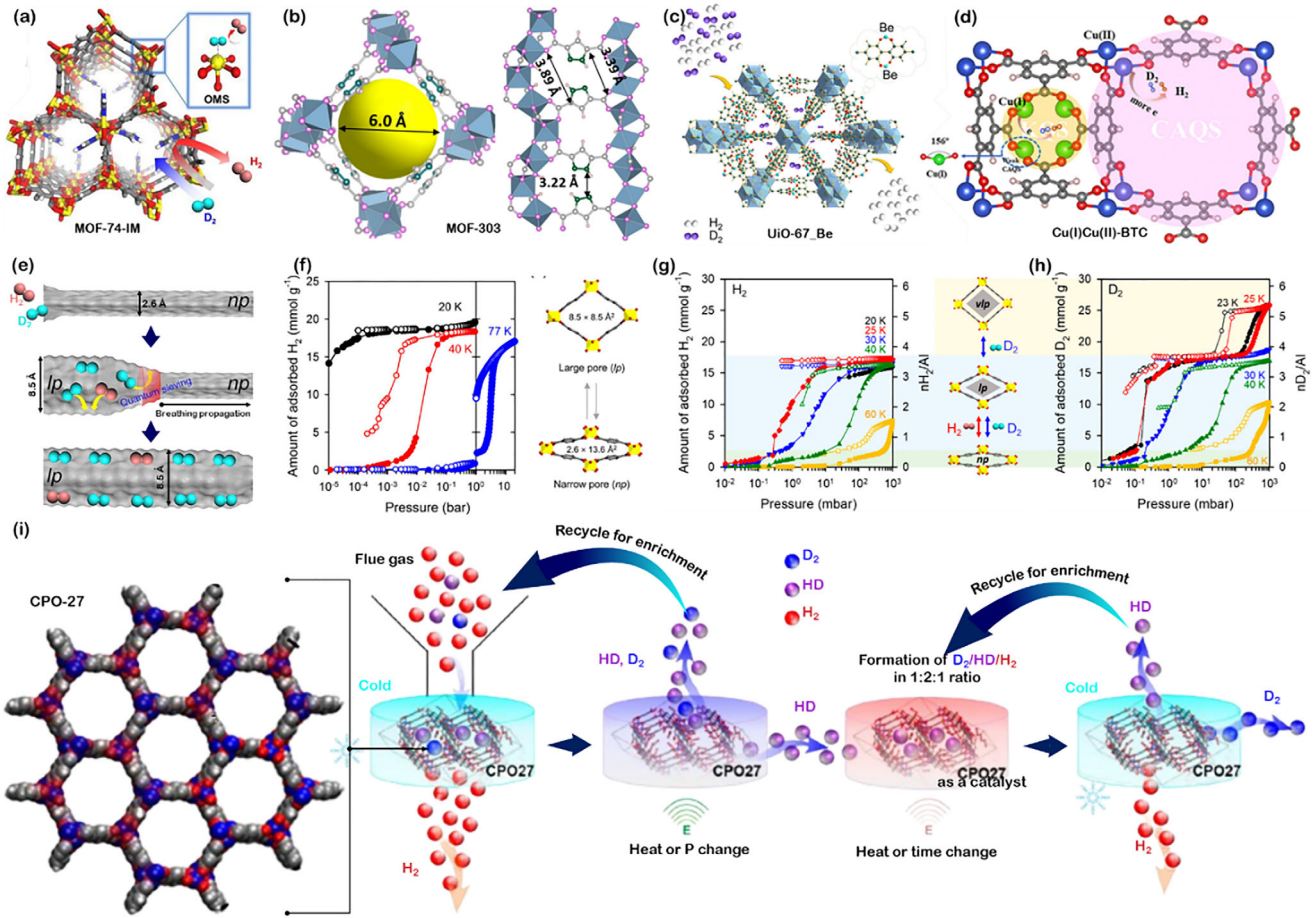
Framework structures of (a) MOF‐74‐IM [197], Reproduced with permission [197]. Copyright 2017, American Chemical Society; (b) MOF‐303 [213], Reproduced with permission [213]. Copyright 2023, Elsevier B.V.; (c) UiO‐67_Be [219], Reproduced with permission [219]. Copyright 2023, American Chemical Society; and (d) Cu(I)Cu(II)‐BTC [203], Reproduced with permission [203]. Copyright 2023, American Chemical Society, highlighting representative porous frameworks relevant to hydrogen isotope separation. (e) Schematic of D_2_ separation via diffusion and structural breathing in MIL‐53(Al). (f) H_2_ adsorption isotherms of MIL‐53(Al) at cryogenic temperatures (20–77 K), illustrating pore‐dependent adsorption behaviour. (e,f) Reproduced with permission [180]. Copyright 2017, American Chemical Society. (g,h) H_2_ and D_2_ adsorption–desorption isotherms of MIL‐53(Al) at various temperatures, showing structural transitions between narrow‐pore, large‐pore, and very large‐pore phases. (g,h) Reproduced with permission [181]. Copyright 2020, American Chemical Society. (i) Schematic of multi‐cycle catalytic enrichment of deuterium to produce HD. Adopted with permission [182]. Copyright 2014, American Chemical Society.

The KQS effect can achieve high selectivity through optimisation of the pore size but is limited to extreme cryogenic temperatures (20–70 K). However, the lack of high affinity adsorption sites could make these adsorbents easier to regenerate after adsorption which would be better suited for large scale separation processes. Oh and co‐researchers [213] explored MOF‐303 (Figure 12b), with a one‐dimensional ultra‐narrow micropore structure. As the exposure pressure increased, the synergy between different mechanisms, including partial D_2_ condensation, became more pronounced, further improving D_2_ uptake.

Noguchi et al. [220]. reported adsorption hysteresis in the H_2_ and D_2_ isotherms for CuBOTf at 40 K attributed to a pore‐blocking effect. This blocking effect was more significant for H_2_ than for D_2_, resulting in a selectivity range of 2.6–5.8 for D_2_ over H_2_ at 40 K. The research led by Oh [179, 180, 181, 182, 191, 197, 199, 213, 221, 222, 223, 224, 225, 226] and Moon [178, 184, 192, 197] have explored various microporous frameworks, including MOFs, COFs, and ZIFs, discovering that the D_2_/H_2_ separations were highly influenced by pore size, temperature, and pressure. They concluded that materials with narrow pores (3.0–3.4 Å) exhibited enhanced D_2_ adsorption due to confinement effects. Figure 12e,f highlight breathing transitions in MIL‐53(Al), a flexible MOF, dynamically shift its structure between narrow pore (np) and large pore (lp) phases [180]. Using these dynamic pore changes, a D_2_/H_2_ selectivity of 13.6 at 40 K was achieved by optimising temperature, pressure, and exposure time. Using the same flexible MIL‐53(Al) MOF they reported the first isotope specific structural transition only triggered by D_2_ and not H_2_ (Figure 12g,h). At 23–25 K, D_2_ caused a second, unexpected transition at ∼200 mbar adsorbing an additional 7.9 mmol g^−1^, suggesting the formation of a novel ‘very large pore’ (vlp) phase (Figure 12h) [181].

Cu zeolitic imidazolate framework (ZIF) has narrow 0.24 nm channels which expand on heating. Jung showed that D_2_ had higher mobility through Cu ZIF than H_2_ even at higher temperatures of 150 K. Nam et al. [227] controlled the tortuosity of the ZIF structure by modifying the ratio of pores of 0.34 nm and 0.17 nm, and found that D_2_/H_2_ selectivity could be increased as a function of the pore tortuosity. He et al. [210] investigated Metal‐organic cages found that the local flexibility in the structure enabled higher uptake and selectivity for D_2_. Bondorf et al. [211] investigated MOF DUT‐8(Ni) which has a flexible structure which has a closed pore and open pore configurations. They found the pores remained closed when exposed to H_2_ at 20 K but opened when exposed to D_2_ at 23.3 K at pressures above 20 kPa.

MOFs have been shown to have some potentially useful properties for hydrogen isotope separation at small scale. However, one of the challenges is producing these adsorbents at larger scales as required for practical separation. Many MOFs can be difficult to produce and require high temperatures and pressures (solvothermal conditions) with very low space time yields. Alternative methods have been developed to improve yield and reduce reaction time by providing additional energy input for instance microwaves, electrochemical, sonochemical and mechanochemical synthesis. Of these, electrochemical synthesis has been used by BASF to produce MIL‐100 and HKUST‐1 at commercial scales with space time yields of 2000 kg m^−3^ d^−1^ [228]. Production of some MOF types such as MOF‐74(Ni) have been shown to be possible under aqueous conditions either at ambient [229] or reflux conditions [230, 231] with high space time yields of 1284 kg m^−3^ d^−1^. Another concern with MOF scale up is the chemical and thermal stability. Many MOF types especially those with open metal sites such as MOF‐74 and HKUST‐1 are susceptible to degradation from exposure to H_2_O and other contaminant gases which is difficult to avoid during column installation and after multiple column cycles. Thermal treatment is used to remove contaminants which is vital for tritium handling processes. However, MOFs tend to have low thermal stability due to their organic component and so this make them more difficult to use even when normal operation is at cryogenic temperatures.

MOFs are still at an early stage of development and as such production at scale is still an issue that needs to be solved. This is made more difficult by the wide varieties of MOFs of interest which may all need their own reaction scale up studies. Other scale‐up issues that need to be investigated are pelletization and heat management especially with high heat of adsorption MOFs and MOFs that require extreme low temperatures and/or pressures.

#### 5.2.3 Tritium Radiation Stability of Adsorbents

One potential concern of using adsorbents in high tritium applications is their radiation stability. Recently researchers have tested both MOFs and zeolites under different types of radiation to mimic long term exposure to tritium as summarised in Table 3. For comparison of the radiation conditions to tritium, if it is assumed that an adsorbent has a T_2_ uptake of 1 mmol g, the irradiation dosage from a year of constant use for T_2_ separation can be calculated:

(2) $$\begin{array}{c} \begin{array}{c} Tritium\;decay\;heat\;\times Tritium\;content\;\times Time\;\left(s\right)\\ 1.944\times 1\;\times 31536000=60\;MGy\;per\;annum \end{array} \end{array}$$

**TABLE 3:** Irradiation tests on the stability of MOFs and zeolites.

Adsorbent	Adsorbent type	Irradiation type	Irradiation dosage (MGy)	Result	Refs.
SSZ	Zeolite	β (electron beam irradiations	1–9	No effect	Kumar et al. [212]
MOF‐303	MOF	β‐irradiation (Dreamy electron gas pedal)	0.005–1	No effect	Huang et al. [232]
UiO‐66 (Hf, Zr, Th, Pu or Ce)	MOF	γ (Co60 source) and α radiation (5 MeV He^2+^ ions)	1–85	Loss of crystallinity and BET surface area	Hastings et al. [233]
UiO‐66(Pu)	MOF	Self‐irradiation	6		

Hastings et al. [233] tested the radiation stability of different metal nodes in UiO‐66 including thorium and plutonium using gamma radiation and alpha radiation. They correlated the radiation stability with the strength of the bond between the metal node and the organic linker. The metal node was found to adsorb the ionising radiation energy and so the bond strength was an important factor in the overall stability. They detected loss of crystallinity and reduction in BET surface area after exposure to radiation. Tritium not only introduces radiation‐based stability issues but it can isotope exchange with protium in the framework and decay to helium. However, to the best of our knowledge, there are currently no published studies evaluating the long‐term stability of MOFs under realistic tritium exposure conditions. Studies on tritium‐exposed polymers have reported protium off‐gassing, indicating hydrogen isotope exchange and radiolytic degradation of organic components [234]. These observations suggest that similar degradation mechanisms may occur in porous organic framework materials, including MOFs and COFs, owing to the presence of organic linkers. Consequently, the long‐term structural stability and tritium compatibility of these materials remain important unresolved challenges that require systematic investigation before their deployment in fusion fuel cycle applications.

#### 5.2.4 Harnessing Zeolite Stability for Efficient Hydrogen Isotope Separation

The well‐established properties of zeolites, particularly their exceptional chemical and thermal stability, cannot be overlooked in this context. As rigid, stable porous materials, zeolites provide a robust framework suitable for atomic‐scale metal loading, which is especially advantageous in tritium‐related applications where reducing tritium inventory is critical for safety and efficiency. Their uniform pore structures and durability under extreme conditions make them highly reliable for long‐term use in isotope separation systems. Studies on zeolites or porous adsorbents show that heavier isotopes are preferentially adsorbed over lighter ones due to the KQS effect [201, 235, 236, 237, 238, 239, 240]. Interestingly, this isotope‐dependent adsorption behaviour appears largely independent of pore size and cation size, with few exceptions linked to the chemical affinity of silver [185, 241], and copper [242, 243] cations that are swapped in to replace other metal ions in the zeolite aluminosilicate framework.

Commercial and ion‐exchanged zeolites, including Chabazite (CHA) [163, 187, 244, 245], Zeolite Rho (RHO) [246, 247], Linde Type A (LTA) [157, 160, 161, 162, 248, 249, 250, 251], Zeolite Socony Mobil‐Five (MFI) [242, 252, 253], Mordenite (MOR) [188, 254, 255, 256, 257, 258, 259], and Faujasite (FAU) [161, 185, 189, 241, 246, 260, 261, 262, 263, 264, 265], have been extensively studied for hydrogen isotope separation. It has been observed that cations with higher charge densities tend to exhibit the strongest thermodynamic selectivity. Furthermore, the intriguing ‘trapdoor’ phenomenon in zeolites, first reported in Cs‐exchanged chabazite [244] is shown to selectively adsorb D_2_ at low concentrations at much higher temperatures than other adsorbents. Recently trapdoor behaviour has been reported in other zeolites. Yu et al. [253] found that in ZSM‐25, Na^+^ cations blocked H_2_ and D_2_ adsorption while K^+^ cations lead to high uptake and selectivity at 77 K. ZSM‐25‐Na shows temperature dependent trapdoor behaviour with peak uptake and selectivity at 195 K. GIS type zeolite also appears to show trapdoor behaviour [243]. The underlying mechanism driving enhanced selectivity of the trapdoor zeolite remains an area of active investigation, underscoring the need for further studies to unlock the full potential of zeolites in this field.

Many zeolites contain exchangeable cations which can be exchanged and so alter the chemical affinity driving force for hydrogen isotope separation through CAQS. Zhang et al. [185] explored this concept by investigating the chemical affinity of Ag‐exchanged Zeolite Y for separating hydrogen isotopes, as depicted in Figure 13a. The Ag^+^ sites preferentially absorb heavier hydrogen isotopes, with the highest D_2_/H_2_ selectivity of 10 observed at 90 K and lead to enhanced uptake. This high density of adsorption sites, combined with their strong interaction with hydrogen isotopologues, significantly enhances the adsorption performance and selectivity of the material. DFT calculations and thermal desorption spectroscopy (TDS) suggest Ag sites have potential for D_2_/H_2_ selectivity at higher temperatures.

**FIGURE 13:**
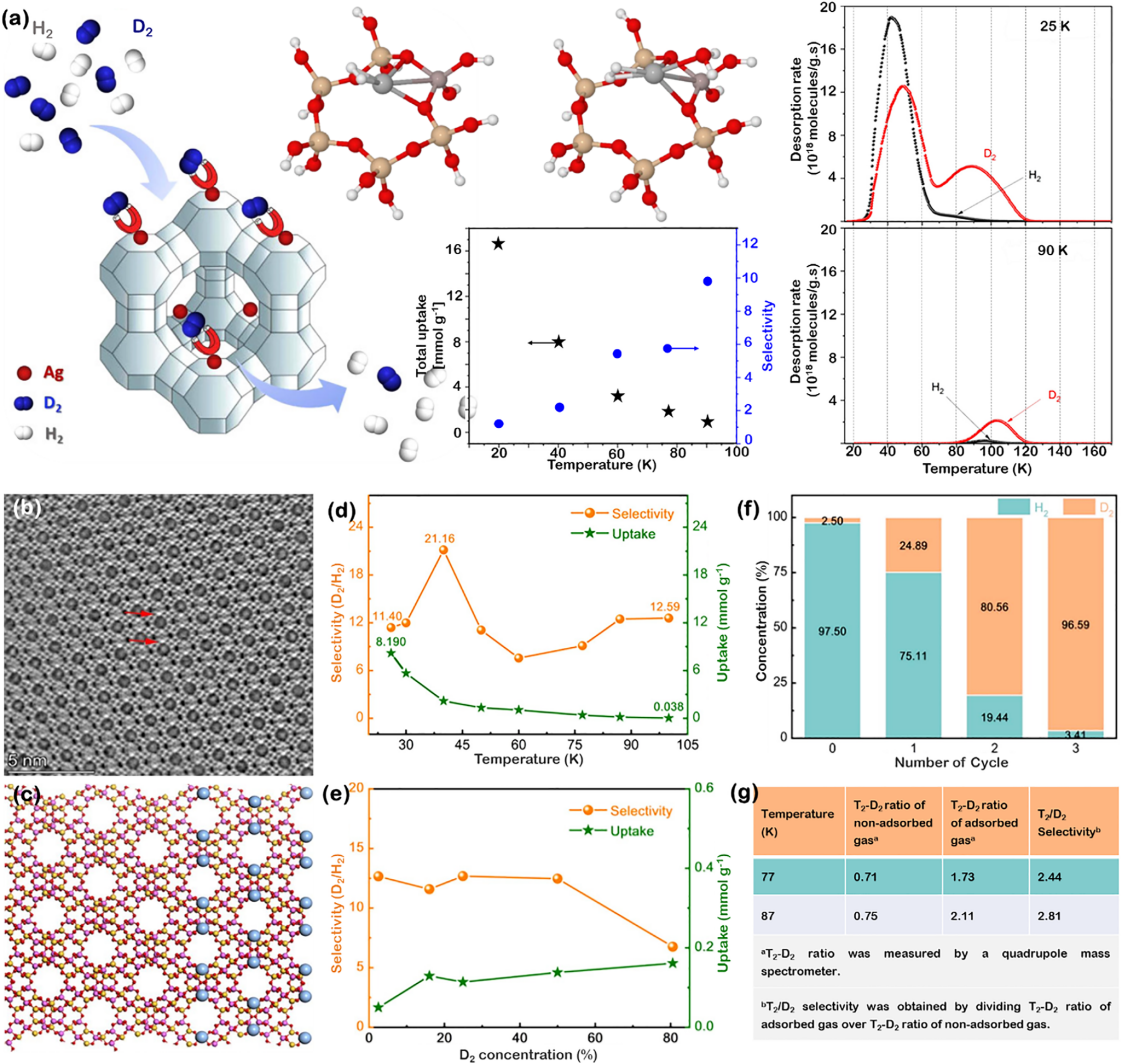
(a) Schematic representation of Ag‐exchanged FAU zeolite for efficient hydrogen isotope separation, along with desorption rate plots of H_2_ and D_2_, uptake amounts, and selectivity at various temperatures. Reproduced with permission [223]. Copyright 2022, American Chemical Society. (b) Atomic‐resolution imaging of Ag(I)Y using iDPC‐STEM. (c) Simulated iDPC‐STEM image depicting the crystal framework of zeolite Y along the [110] direction. (d) D_2_/H_2_ selectivity and corresponding uptake values as a function of exposure temperature for Ag(I)Y zeolite. (e) D_2_/H_2_ selectivity and uptake values as a function of D_2_ concentration, ranging from 2.5% to 80.56%, at 87 K for Ag(I)Y zeolite. (f) Cyclic studies of D_2_ enrichment through temperature swing cycles, where Ag(I)Y is exposed to a D_2_‐H_2_ mixture containing 2.5% D_2_ at 1.0 kPa and 87 K for 30 min. (g) T_2_/D_2_ selectivity of Ag(I)Y measured at 77 and 87 K. (b–g) Reproduced with permission [241]. Copyright 2024, Elsevier B.V.

Some of the only tritium adsorption measurements in the public domain have been performed by Song et al. [241] who systematically investigated the adsorption selectivity of hydrogen isotopes (H_2_, D_2_, and T_2_) on Ag(I)‐exchanged zeolite Y (Ag(I)Y) using TDS) and a quadrupole mass spectrometer (QMS) (Figure 13b–g). Their results were further compared with those obtained for Cu(II)‐exchanged zeolite Y, NaY, and HY zeolites, providing valuable insights into the performance differences among these materials. More importantly, they were the first to report separation experiments of D_2_ and T_2_ using Ag(I)Y, employing the CAQS mechanism. This study represents a significant breakthrough as it successfully achieved and quantified the T_2_/D_2_ selectivity, which had not been reported before. This study also highlights that a significant decrease in T_2_/D_2_ selectivity was observed when compared to D_2_/H_2_ selectivity under similar conditions. This reduction can be attributed to the smaller relative mass difference between T_2_ and deuterium D_2_ compared to that between D_2_ and H_2_.

Further testing of Ag(I)Y have been done on its D_2_ enrichment capabilities using multiple temperature swing cycles (Figure 13f). In the first cycle, Ag(I)Y was exposed to a 1.0 kPa hydrogen mixture containing 2.5% D_2_ at 87 K for 30 min. In the second cycle, a hydrogen mixture with 24.89% D_2_ was introduced, and 80.56% D_2_ enrichment was achieved. After three cycles, the D_2_ concentration reached 96.59%. These findings demonstrate that Ag(I)Y is effective for extracting heavy hydrogen isotopes from a diluted mixture with a high H_2_ concentration using a temperature swing process.

Mechanisms in zeolites such as chemical affinity and flexible structures have been proven to achieve high hydrogen isotope selectivity. Combined with the high structural stability and tritium compatibility of zeolites, they are currently one of the most promising adsorbents for use in the tritium fuel cycle. However, adsorption separation of hydrogen isotopes using zeolites is still at an early stage of development and most of the data is limited to small scale equilibrium adsorption experiments. From these experiments there are no clear trends between framework type, pore size, cation and selectivity which makes predicting behaviours and modelling difficult (Figure 14). There still needs to be more work and consideration of how well these adsorbents can scale‐up to be used in adsorption columns and the performance of these adsorbents in temperature‐swing or pressure‐swing operation.

**FIGURE 14:**
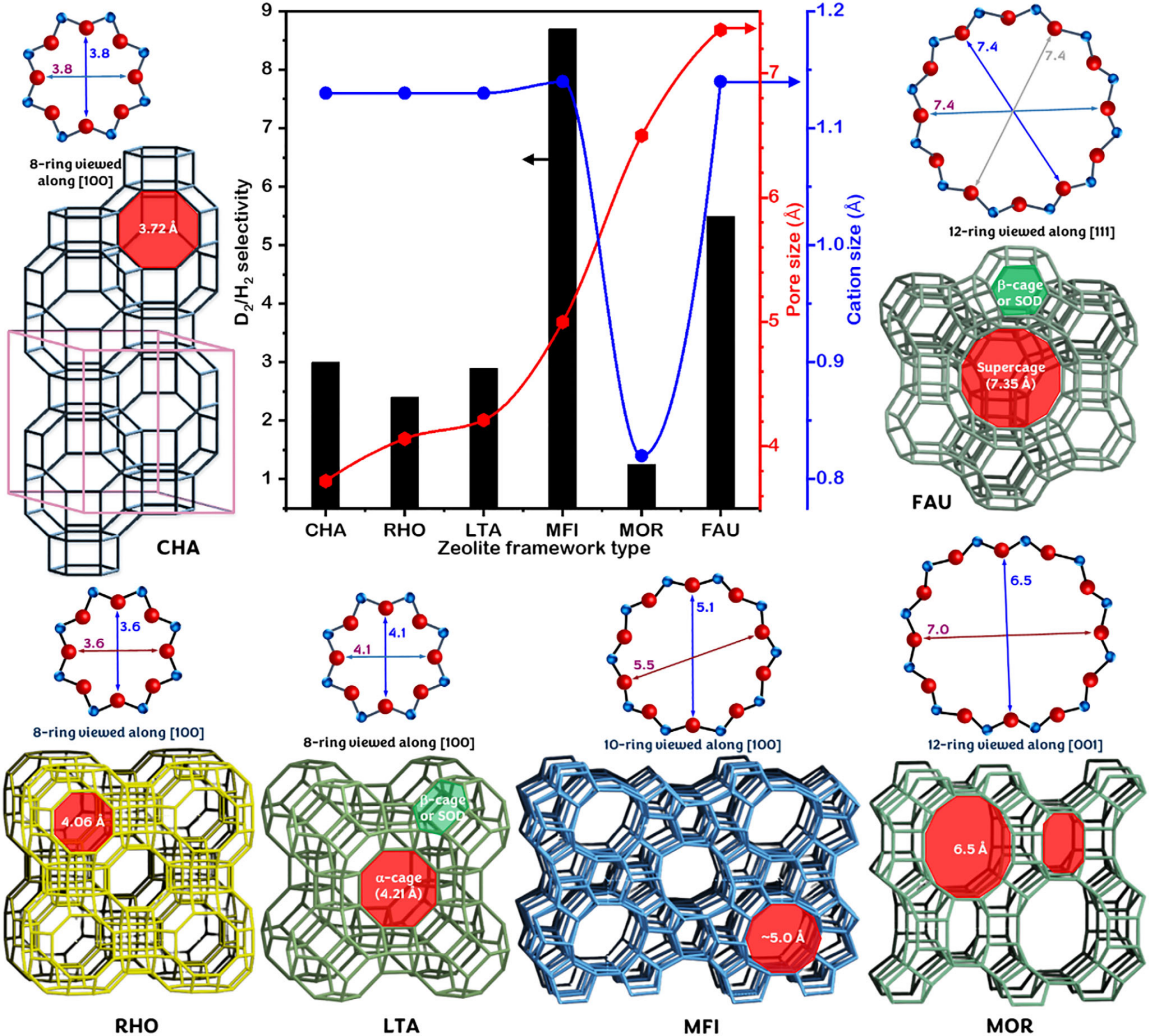
Adsorption performance of various zeolites (Chabazite (CHA) [187], Zeolite Rho (RHO) [246], Linde Type A (LTA) [250], Zeolite Socony Mobil‐Five (MFI), Mordenite(MOR) [254], and Faujasite (FAU) [185]) for hydrogen isotope separation at 77 K, represented as D_2_/H_2_ selectivity (column graph). The figure also includes the framework structure and pore structure of each reported zeolite, along with plots of the cationic size of exchangeable cations and the pore opening size of zeolitic pores, highlighting their influence on selectivity.

### 5.3 Porous Carbon Materials

Apart from metal‐organic frameworks and zeolites, carbon‐based adsorbents have garnered significant attention for hydrogen isotope separation due to their highly developed porosity, large surface area, and high surface reactivity [178, 200, 202, 222, 224, 226, 266, 267, 268, 269, 270, 271, 272, 273, 274, 275, 276, 277, 278]. These materials offer additional advantages, including cost‐effective mass production from inexpensive precursors such as agricultural or biowaste and relatively straightforward manufacturing processes, making them easily scalable. Various forms of carbon‐based adsorbents, including amorphous porous carbons, carbon nanotubes, graphene, and so on, have been explored as promising candidates for efficient hydrogen isotope separation. In this section, we provide an overview of the potential applications of carbon‐based adsorbents in hydrogen isotope separation, highlighting their advantages, mechanisms, and recent research advancements (Figure 15).

**FIGURE 15:**
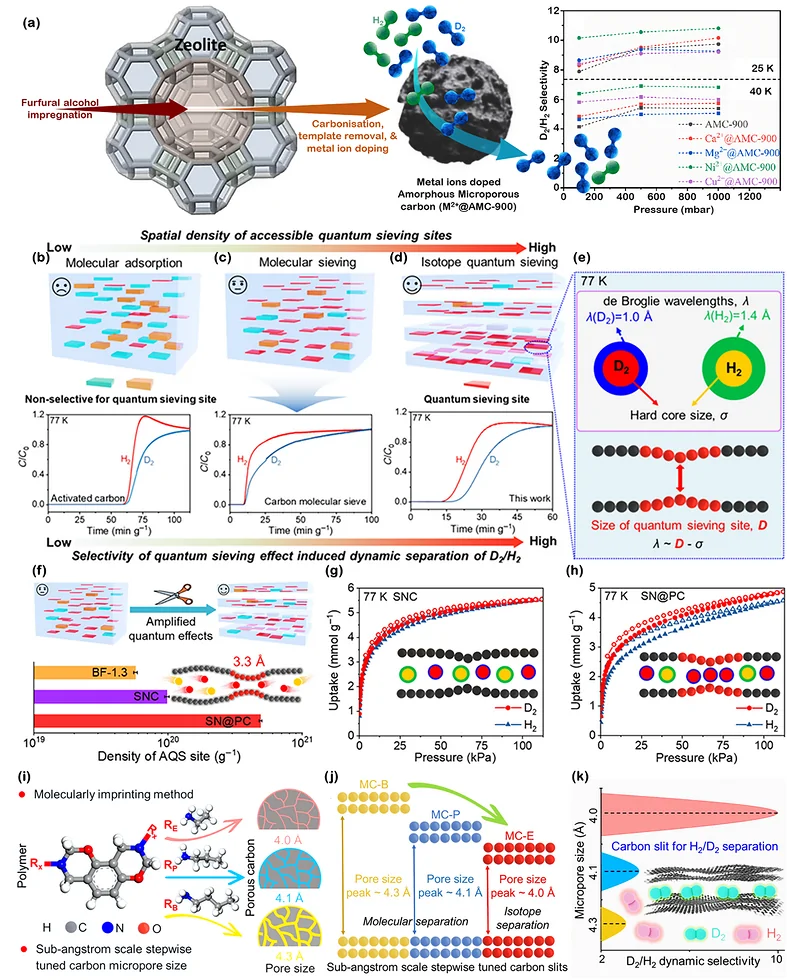
(a) Schematic of the synthesis of metal‐doped amorphous microporous carbon (M^2+^@AMC) and its D_2_/H_2_ separation performance at cryogenic temperatures. Reproduced with permission [222]. Copyright 2024, Elsevier B.V. (b–d) Comparison of AQS sites in activated carbons, carbon molecular sieves, and two‐dimensional carbon molecular sieves, together with representative D_2_/H_2_ breakthrough performance at 77 K. (e) Schematic illustrating the relationship between hydrogen isotope de Broglie wavelength and carbon slit size for quantum sieving. (f) Comparison of accessible 3.3 Å micropore density in SN@PC, SNC, and commercial CMS BF‐1.3. (g,h) D_2_/H_2_ adsorption–desorption isotherms of SNC and SN@PC at 77 K. (b–h) Reproduced with permission [267]. Copyright 2024, American Institute of Chemical Engineers. (i) Schematic of polymer precursor design and molecular imprinting strategy for sub‐angstrom carbon nanoslit tuning. (j) Illustration of the transition from molecular sieving to quantum sieving with decreasing slit size. (k) Dynamic D_2_/H_2_ separation performance of carbon nanoslits with an optimum slit width of approximately 4.0 Å. (i‐k) Reproduced with permission [266]. Copyright 2025, Elsevier B.V.

Oh and his research team [222] explored the effects of metal doping on pore‐size modulation and binding affinity improvement. The study employed a straightforward impregnation method to prepare amorphous microporous carbons (AMC), using ammonium‐form zeolite Y (NH_4_Y) as the template and furfuryl alcohol (FA) as the carbon source. The synthesized AMC was further modified through doping with various metals (Ca^2+^/Mg^2+^/Ni^2+^/Cu^2+^). Metal doping‐induced pore modulation significantly improved the D_2/_H_2_ selectivity with the highest selectivity of 10.81 observed at 25 K and 1000 mbar for Ni^2+^@AMC‐900 (Figure 15a). This result highlights the strong potential of nickel as a dopant for enhancing CAQS effects.

In 2024, Wang and colleagues [267] developed a method to create and expose a high density of accessible quantum sieving (AQS) sites in two‐dimensional (2D) porous carbon adsorbents (SN@PC) (Figure 15b–h). The selective enrichment of AQS sites was attributed to the polymer coating on SN@PC, which, after thermal treatment, formed narrow micropores with size distributions cantered at 3.3 Å. These micropores intensified the KQS effect, with SN@PC demonstrating an 8.5‐fold increase in AQS site density and a 6.7‐fold improvement in D_2_​ diffusion rate over commercial carbon molecular sieves with a selectivity of 4.6 at 77K. They also reported that Aspen simulations of pressure swing adsorption indicated that D_2_​ product purity could reach 93.1% after 11‐cycle enrichment using a 1% D_2_​/99% H_2_​ mixture as feed.

Very recently Wang et al. [266] reported another novel method for hydrogen isotope separation by precisely tuning carbon nano slits at the sub‐angstrom scale through a thermal‐induced molecular imprinting strategy. This approach produced nano slits (4.0‐4.3 Å) with 4.0 Å slits achieving high D_2_​ adsorption capacity and a dynamic selectivity of 10.1 at 40 K (Figure 15i,k) [181, 199, 203, 213, 224, 226, 248, 266, 279, 280, 281, 282, 283]. Jung et al. [223] reported gating effect in nanoporous carbon prepared from sesame seeds, leading to selectivity of hydrogen isotopes at mild temperature (120 K).

While activated carbon may exhibit some selectivity towards hydrogen isotopes, its overall selectivity remains relatively low compared to other advanced materials designed specifically for isotope separation. Furthermore, factors such as chemical and physical alterations during extended use or under harsh conditions can degrade the adsorption capacity of activated carbon over time, reducing its long‐term applicability.

## 6 Sorbent Materials for Cryogenic Gas Chromatography

There have been a few recent reports of composite porous materials synthesized and implemented in cryogenic gas chromatography experiments. They contain alumina as a strong support for a MOF or other porous material that exhibits the KQS effect, they also make use of metal ions for their strong selectivity.

In 2006, Junbo et al. [38], investigated the separation of hydrogen isotopes using gas chromatography with a combined column consisting of a 5 Å molecular sieve and Al_2_O_3_ under cryogenic conditions. In their study, the 5 Å molecular sieve and Al_2_O_3_ were used as alternatives to the costly noble metal palladium. Key benefits of the setup include its simple design (Figure 16), low operational costs, and minimal investment, making it highly suitable for laboratories and facilities requiring small amounts of high‐purity hydrogen isotopes.

**FIGURE 16:**
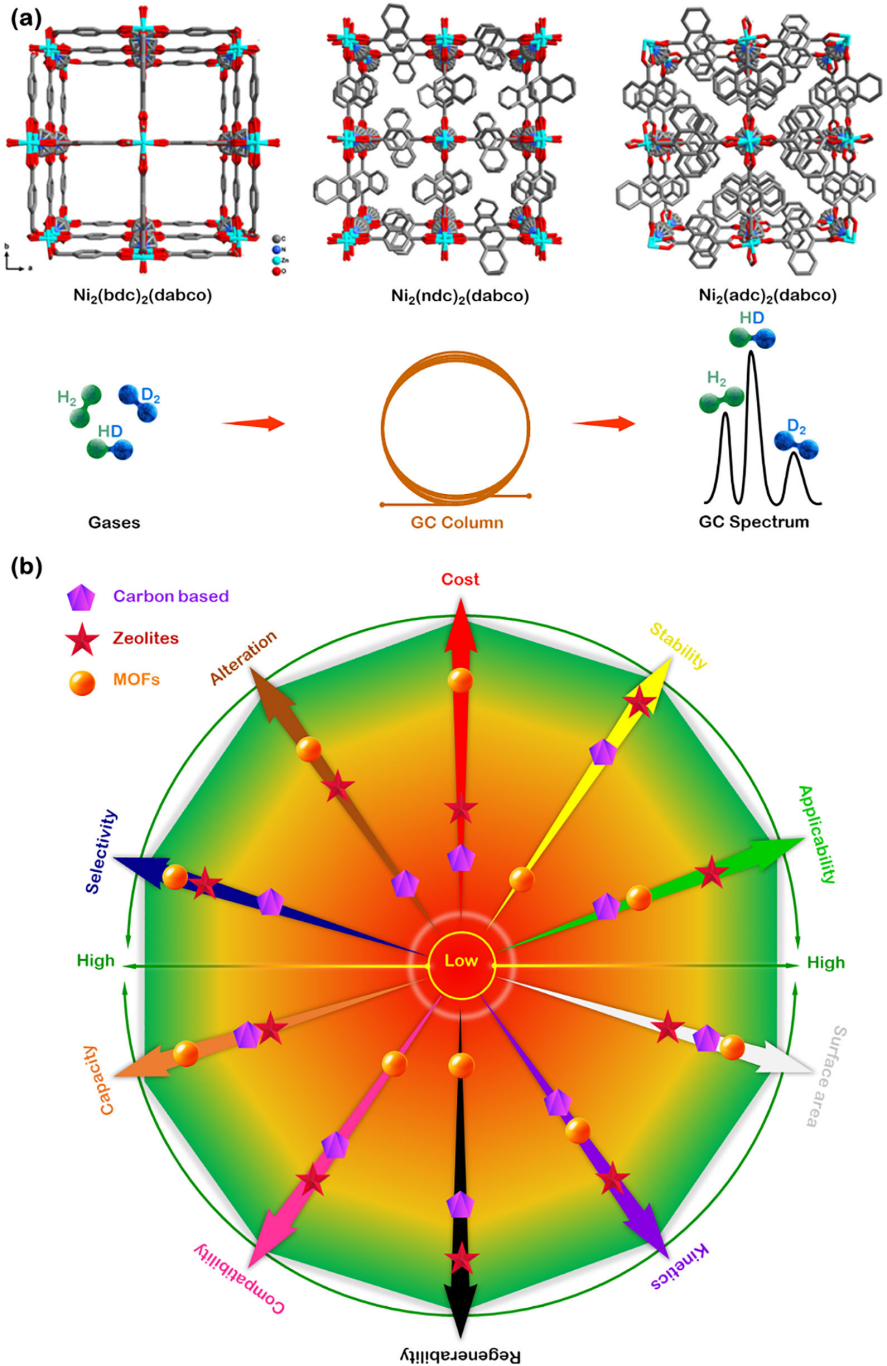
(a) Crystal structures of Ni_2_(L)_2_(dabco) derived from isomorphous Zn_2_(L)_2_(dabco) [286, 287, 288], together with a schematic of cryogenic gas chromatographic separation of H_2_, HD, and D_2_ using a Ni_2_(L)_2_(dabco)@γ‐Al_2_O_3_ composite stationary phase [285]. Adopted with permission [285]. Copyright 2022, Elsevier B.V. (b) Comparative assessment of zeolites, MOFs, and carbon‐based materials for hydrogen isotope separation based on different performance‐related attributes. The comparison is intended to provide a qualitative overview of the general characteristics of each sorbent class based on representative literature reports. It should not be interpreted as a direct quantitative comparison or ranking, as the reported performance is highly dependent on material formulation (including precursor selection and synthesis strategy), activation and post‐synthetic modification, the experimental methodology employed, etc.

Yang et al. [121] demonstrated hydrogen isotope separation using CGC with a novel stationary phase material, MnCl_2_@CPL‐1@γ‐Al_2_O_3_. This material combines the selective adsorption capability of manganese chloride (MnCl_2_), the kinetic quantum sieving effect of the metal‐organic framework (MOF) compound CPL‐1, and the structural stability of γ‐Al_2_O_3_. To fabricate the novel stationary phase materials, CPL‐1 was loaded onto γ‐Al_2_O_3_ particles (80‐100 mesh) via a repeated in situ crystallization process, followed by the impregnation of MnCl_2_ onto CPL‐1@γ‐Al_2_O_3_. The column provided a good separation resolution (*R* = 1.62) and produced Gaussian‐like chromatographic peaks.

Furthermore, in their study, Kong et al. [284] prepared Ni‐4PyC@γ‐Al_2_O_2_ composite material using a liquid‐phase epitaxy method. This composite was then utilized for D_2_/H_2_ separation in a 0.6 m × 2 mm chromatographic column at 77 K. Under optimal conditions, the separation achieved a resolution of 1.84 with a separation time of 7.47 min. Li et al. [285] reported the synthesis of a series of pillared‐layer MOFs, Ni_2_(L)_2_(dabco), with the highest enrichment factor for D_2_ over H_2_ of 6.8 achieved in static adsorption experiments. Figure 16a illustrates the crystal structures of Ni_2_(L)_2_(dabco). Additionally, it presents a schematic of cryogenic gas chromatographic separation of hydrogen isotopes (H_2_, HD and D_2_) utilising Ni_2_(L)_2_(dabco)@γ‐Al_2_O_3_ as the column material (stationary phase). The synergy of the MOF structure and the γ‐Al_2_O_3_ support enables efficient isotope separation, driven by enhanced adsorption selectivity and kinetic sieving effects at cryogenic temperatures. These findings underscore its potential as an effective stationary phase for hydrogen isotope separation.

## 7 Summary and Outlook

Efficient separation of H_2_, D_2_, and T_2_, is critical for optimising the tritium fuel cycle in fusion reactors, with growing importance as fusion technologies progress globally. Hydrogen isotope separation using sorbents is one of the most promising techniques due to the very high isotope selectivity achieved by many different sorbents. Palladium is one of the best options for a separation sorbent, it provides separation at amenable temperatures and there is the potential for optimisation of the sorption kinetics via tailored facets and enhanced stability via porous supports. Metal hydrides are suitable for chromatographic processes however thermal treatments may be needed for regeneration.

Adsorption based separation is still at a very early stage of development compared to cryogenic distillation, membranes and TCAP, more data is required. Most studies have focused primarily on the separation of H_2_ and D_2_, with very few addressing the separation of D_2_ and T_2_, an area that requires further attention. Furthermore, most reported adsorbents can only achieve high selectivity at cryogenic temperatures and may require temperature‐swing operation to regenerate them due to their high affinity for hydrogen. This could be a severe drawback for scale up as temperature changes of large adsorption columns can be very slow and energy intensive, especially at cryogenic temperatures which would require refrigeration. Some adsorbents may be able to operate at higher temperatures or be able to operate in pressure‐swing operation, but more work is required to select adsorbents based on these practical considerations.

The leading materials for hydrogen isotope separation include metal hydrides, metal‐organic frameworks (MOFs), zeolites, and carbon‐based adsorbents. When considering factors such as cost, stability, operational toughness, surface area, kinetics, regenerability, compatibility, capacity, and selectivity, zeolites emerge as the most promising adsorbent (Figure 16b). However, challenges remain related to their low adsorption capacity, selectivity and hysteresis.

MOFs and organometallic porous adsorbents, although promising, still require extensive study, particularly regarding their reactivity to radioactive tritium. As isotope exchange occurs in organic materials when exposed to tritium it's vital to understand the long‐term stability and performance of these materials in a tritium‐rich environment. Looking forward, as the fusion industry grows, the challenge of achieving hydrogen isotope separation with high energy and separation efficiency but low tritium inventory has become more vital. Emerging high performance technology using adsorbents and semi‐continuous Pd systems have shown that achieving this challenge is now possible, providing a path towards fusion as a practical and clean energy source.

## Conflicts of Interest

The authors declare no conflicts of interest.

## Data Availability

The data that supports the findings of this study are available in the supplementary material of this article.
